# Different Reactions Define the Electrochemical Window in 1‐Butyl‐3‐Methylimidazolium Triflate on Gold and Platinum Electrodes

**DOI:** 10.1002/cphc.202500096

**Published:** 2025-10-16

**Authors:** Frederik J. Stender, Marcel Risch

**Affiliations:** ^1^ Institut für Materialphysik Georg‐August‐Universität Göttingen Friedrich‐Hund‐Platz 1 37085 Göttingen Germany; ^2^ Nachwuchsgruppe Gestaltung des Sauerstoffentwicklungsmechanismus Helmholtz‐Zentrum Berlin für Materialien und Energie GmbH Hahn‐Meitner‐Platz 1 14109 Berlin Germany

**Keywords:** 1‐butyl‐3‐methylimidazolium triflate, differential electrochemical mass spectrometer, electrochemical window, ionic‐liquid‐water mixtures, water‐in‐salt

## Abstract

Ionic liquids (IL) make excellent candidates for many energy storage devices due to unique and tunable properties such as a large electrochemical window (ECW). Water as an impurity in 1‐butyl‐3‐methylimidazolium (BMIM) triflate is investigated on platinum and gold electrodes in a stagnant glass cell and in a flow‐cell coupled to a differential electrochemical mass spectrometer (DEMS). It is found that the ECW closes with increasing water content on both gold and platinum electrodes in both setups. Platinum has a smaller ECW than gold, where the difference mainly stems from the limiting reduction reaction, as identified based on DEMS. Below 1.11 M_H2O_/L_IL_, the anodic reaction is predominantly IL decomposition and above the oxygen evolution reaction for both materials. The cathodic limit is given by the hydrogen evolution reaction for platinum independent of water content and gold above 1.66 M_H2O_/L_IL_, while it is IL decomposition below. The study highlights the interplay between electrode material and electrolyte for tailoring the ECW for applications involving intentional or unintentional mixing of water with IL.

## Graphical abstract

Ionic liquids have attractive features such as a large electrochemical window. It is shown that it depends on both the electrolyte and electrode when water is added to an imidazolium ionic liquid.

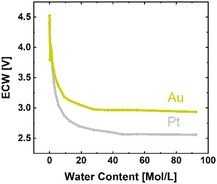

## 1 Introduction

Ionic liquids (ILs) are organic salts that are liquid at or close to room temperature with tunable characteristics depending on the used cation and anion. They gained increased interest in academic research as well as industry for use in, for example, synthesis and catalysis,^[^ ^1^ ^]^ supercapacitors,^[^ ^2^ ^]^ and batteries.^[^ ^3^ ^]^ The increasing interest stems from the unique properties ILs have to offer in comparison to many used organic solvents regarding tunability of their physicochemical properties, for example, melting point, viscosity, conductivity, toxicity, chemical stability, solubility of solvents and gases, and the electrochemical window (ECW).^[^ ^4^, ^5^ ^]^ Therefore, they are also called designer solvents. A further unique property for liquids is that some ILs have negligible sometimes not measurable vapor pressure reducing the environmental exposure.^[^ ^6^ ^]^

The ECW of ILs can exceed 6 V,^[^ ^7^ ^]^ which surpasses mixtures of organic solvents and salts used in electrochemical devices. This potentially offers improvements in the energy density of energy storage devices by accessing uncommonly high or low oxidation states. However, the ECW as well as most other properties depend highly on impurities in the IL. The most common impurity is water, which is taken up from air and often binds strongly to the ILs. For the imidazolium‐based ILs, simulations predict the formation of a rather complex structure in the IL‐water structure, which depends on the ions as well as the water content.^[^ ^8^, ^9^, ^10^ ^]^ Creating either chains of water and anions bonded by hydrogen bonding or clusters of water separated by the ions at higher water contents. The rather strong bonding of water makes it hard to remove small amounts of water. A commonly used approach is exploiting the nonexisting vapor pressure and drying the IL in a vacuum for multiple hours.^[^ ^11^, ^12^, ^13^ ^]^ How strong the water is bound depends on the ability to create hydrogen bonding between the anion and water,^[^ ^14^ ^]^ which also determines the miscibility with water. However, also the chain length of the imidazolium cation clearly influences the hydrophobicity of the IL; the longer the chain length, the lower the maximum water content before phase separation.^[^ ^15^ ^]^

The limits of the ECW of the dry ILs are often assigned to the highest occupied molecular orbital (HOMO) for the oxidation of the anion at the anodic limit and to the lowest unoccupied molecular orbital (LUMO) for the reduction of the cation at the cathodic limit.^[^ ^16^ ^]^ However, it was predicted that the interaction of the ions among each other stabilizes the ions and increases the ECW.^[^ ^17^ ^]^ Simulations further predict that the cation may sometimes be the limiting factor for the anodic and cathodic limits in the imidazolium‐based ILs.^[^ ^17^ ^]^ The addition of water introduces an additional component, with a much narrower gap between the HOMO and LUMO levels, making it a candidate for the limiting reactant of both potential limits. However, water also changes the preferred distance and sometimes the position between the anion and cation due to the aforementioned cluster and chain building.^[^ ^8^ ^]^ This may influence the effective HOMO and LOMO levels either by distance or bonding environment, which may lead to a different ECW of the IL due to destabilization. While the effect of water on the ECW of ionic liquids was previously investigated in the context of humidity,^[^ ^18^, ^19^ ^]^ the effect of defined steps of added water as well as the decomposition products to investigate the limiting reactions is scarce. Mass spectroscopy under UVH conditions showed that water oxidation occurred at different potentials on Pt, Au, and Pd, while the oxidation of the triflate stayed at the same potential.^[^ ^13^ ^]^

In this work, we used 1‐butyl‐3‐methylimidazolium triflate [BMIM][OTf] from the well‐known and investigated family of the imidazolium‐based ILs due to their ability to be miscible with water to a high extent (with a carefully chosen anion). The IL is liquid at room temperature (melting point 18 °C^[^ ^20^ ^]^). The goal of our study is a better understanding of the reactions limiting the ECW of the IL‐water mixture, which is investigated by determining the limiting potentials with systematically increasing water content as well as by measuring the reaction products on gold and platinum using differential electrochemical mass spectroscopy (DEMS). Platinum and gold were chosen because they have a large stable window and have been investigated previously for ILs,^[^ ^13^, ^21^ ^]^ and platinum is known to reduce water to hydrogen more efficiently than gold, making them good choices for a systematic study where water is expected as a limiting reaction on platinum but not on gold.

## 2 Results and Discussion

### 2.1 Electrochemical Impedance

**Figure 1:**
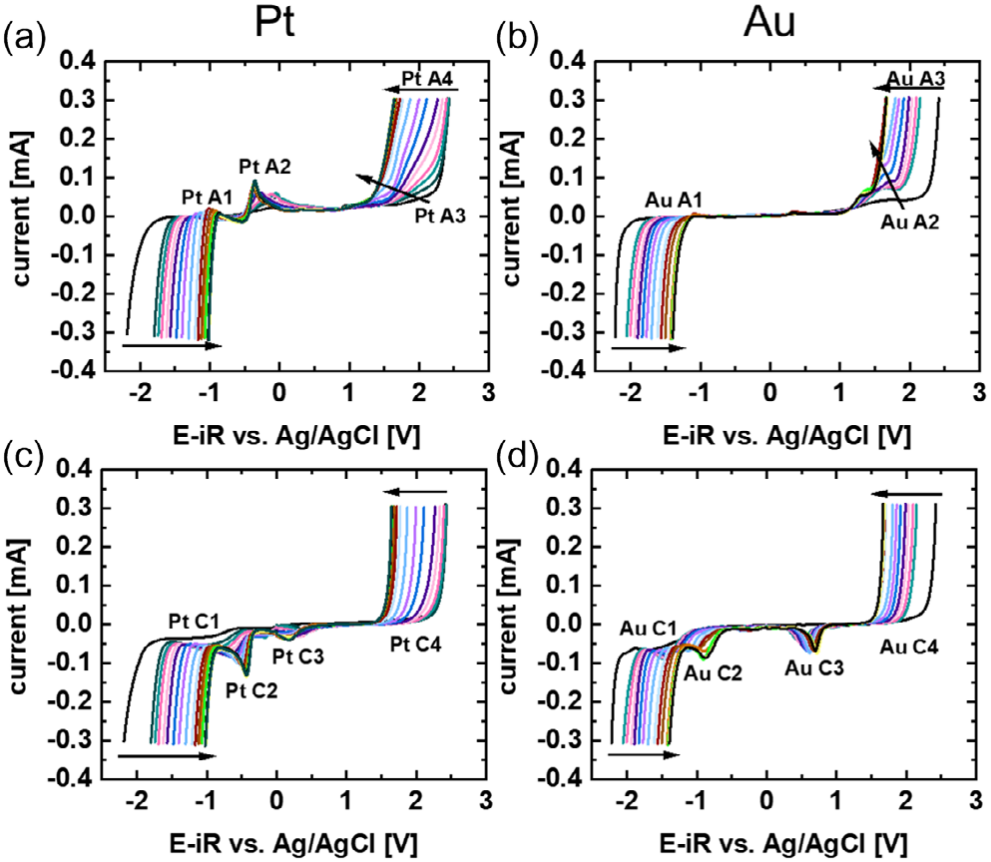
a,b) Anodic and c,d) cathodic scan direction of the 4th cycle of the CV investigations on a a,c) platinum and b,d) gold wire in a glass cell. The black arrows indicate the movement of features with increasing water content from 0 to 92.2 M_H2O_/L_IL_. The full water content steps and conversion to v/v% and mole‐fraction may be found in Table S1, Supporting Information.

Ionic liquids typically have resistances higher than aqueous electrolytes, which reduce the applied potentials. Therefore, we started by investigating the resistance of the IL‐water mixtures using electrochemical impedance spectroscopy (EIS). To avoid contributions from unknown reactions, the EIS was conducted at 0.8 V versus REF (glass cell) and 0.65 V versus REF (DEMS cell), where no reaction in the CVs was observed (**Figure** **1**). Surprisingly, the measurements showed a negative real part of the impedance for higher frequencies. We refer to it as negative resistance to improve readability. Negative resistances (Figure S2 and S4, Supporting Information) in EIS are caused by adsorption, passivation, or artifacts of the setup. The first two occur on the low‐frequency side.^[^ ^22^ ^]^ In the high frequency range, an inductance effect and reaction speed of the reference electrode may be the cause of this phenomenon.^[^ ^23^ ^]^ Further evidence for an artifact was gained from the observation that the negative resistance at high frequencies, normally describing the electrode/electrolyte resistance, was not affected during the uptake of water, while the resistance (at lower frequency) typically attributed to the charge transfer decreased. Therefore, it was most probably an artifact coming from a resistive element of the potentiostat or reference electrode creating a negative resistance circuit.

To test this hypothesis, we first replaced the leakless reference electrode with a Pt quasi‐reference electrode (having no junction resistance) in 1‐hexyl‐3‐methylimidazolium triflate with high water content (≈40 v/v%) in the glass cell. In this experiment, no negative resistances were observed at high frequency (Figure S6, Supporting Information). Then, we systematically added resistors between the Pt wire and the potentiostat lead where a deviation from the measurement without added resistance was clearly observed for 5 kΩ added and above in the range 100 kHz to 1 MHz with no change at lower frequencies (Figure S6, Supporting Information). Higher added resistances increased the deviation. The observed behavior of the leakless reference electrode was best described with a 10 kΩ resistor. A resistance <10 kΩ is expected according to the manufacturer (eDAQ), which agreed with our observation. We conclude that the junction resistance of the used leakless reference electrode caused the negative impedance. Since its effect is limited to frequencies above around 100 kHz and the phase closest to 0° was at frequencies about a decade lower, we conclude that the determination of the uncompensated resistance at lower frequencies was not affected by the artifact of negative resistance and was measured as accurately as possible with the used reference electrode.

We show the extracted resistances for the 3‐electrode glass cell in Figure S3, Supporting Information. A strong decrease from 350 to 20 Ω for more than 10 M added water was observed for increasing water content for both electrode materials and setups. A decrease in resistance was previously reported^[^ ^24^, ^25^, ^26^ ^]^ and arises from a higher mobility of the ions when solvated. For the DEMS cell, the observed resistance at low water content was much lower (70–20 Ω) due to the different geometry of the setup (Figure S4 and S5, Supporting Information). We also investigated the effect of increasing water content using a 2‐electrode connection for the DEMS setup (Figure S4 and S5, Supporting Information). This setup revealed a similar trend compared to using 3 electrodes but without negative resistance at higher frequency and roughly doubled resistance (Figure S5b, Supporting Information). The 2‐electrode measurements further corroborated the reference electrode as the source of the negative resistance.

### 2.2 Electrochemical Behavior in a Glass Cell

We investigated the ECW by cyclic voltammetry (CV) of the platinum and gold wire with different water concentrations in BMIM triflate (Figure 1). Tracking the limiting currents of both platinum (Pt A/C 1&4) and gold (Au A 1&3 and C 1&4) showed that the ECW narrows with increasing water content. Yet, additional redox features were also found.

For platinum and lower water content, an oxidative peak (Pt A3) was visible at the flanks of the limiting increase of the current in the anodic scan direction. This oxidation seemed to move to lower potentials with increasing water content. At high water content, the start of the limiting reaction (Pt A4) began before the oxidative reaction (Pt A3). On the cathodic back scan, a small peak (Pt C3) at ≈0.3 V was found, which was influenced by the water content so that the peak position shifted to 0.17 V for the highest water content. This may be the reduction of the oxidized state mentioned above (Pt A3). However, the peak separation between the redox reactions would be more than 1 V, making the reaction electrochemically irreversible. Therefore, we believe that this peak corresponds to the oxygen reduction reaction. This was also indicated by experiments done under oxygen saturation (Figure S7a,c, Supporting Information), which will be discussed below. Platinum showed an additional redox couple (Pt A/C 2) with a halfwave potential of around −0.5 V. The redox peaks were rather spread out but became well defined at higher water contents. We believe that it is not an impurity but rather a surface reaction catalyzed by the platinum with the ionic liquid since this peak does not appear on gold, it is nearly fully reversible, and it seems to sharpen with increasing water content. For single‐crystalline platinum, it was suggested that the imidazolium ion reduces reversibly to 1‐alkyl‐3‐methylimidazol‐2‐ylidene.^[^ ^21^ ^]^

Gold showed an oxidative peak (Au A2) in the anodic scan direction at higher potentials; however, the peak (at ≈1.5 V) started clearly before the onset of the limiting current. While it also moved to a lower potential with increasing water contents, the shift was less as compared to platinum, and the peak was also visible at high water contents. Furthermore, the cathodic scan showed a corresponding reduction peak (Au C3) at 0.48–0.70 V (low water content to high water content). This peak was missing in the dried IL and was previously assigned to gold oxidation and the corresponding gold oxide reduction, being proposed as an indicator to determine the water content.^[^ ^24^ ^]^ Additionally, an irreversible reductive peak (Au C2) was seen at much lower potentials (≈−1.5 to ≈−1 V for higher to lower water content). Due to the irreversibility of this peak, we assume that this reaction corresponds to the ORR from the produced or leaked oxygen that is still present in the electrolyte.

To investigate the effect of oxygen in the electrolyte, the experiment in the glass cell was repeated with dry electrolyte once assembled and measured as described before. Then, the electrolyte was saturated by bubbling oxygen gas into the cell for 45 min. Figure S7, Supporting Information, indicates that oxygen did produce the reduction peaks at −0.9 V versus Ag/AgCl on gold (Figure S7b,d, Supporting Information) and around 0 V versus Ag/AgCl on platinum (Figure S7a,c, Supporting Information) as also observed in Figure 1. Additionally, the oxygen saturation increased the anodic limit for gold but not platinum, while the cathodic limit was lowered for platinum but not gold. An understanding of the latter observation requires specialized experiments beyond the scope of this work. We conclude that the peaks Au C2 and Pt C3 can be clearly assigned to oxygen reduction.

We define the ECW as the voltage range between the exponential rise and decrease in CV. To determine the onset of these exponentials, we evaluated the potentials at a current of 0.05 mA, corresponding to 0.2 mA cm^−2^_geo_ of wire area exposed to the electrolyte. We additionally extracted the window for 0.1 mA (0.4 mA cm^−2^_geo_) and 0.2 mA (Au: 0.8 mA cm^−2^_geo_ Pt: 0.9 mA cm^−2^_geo_) to demonstrate that the trend is the same for different extraction conditions. The non‐normalized current was used instead of the normalized current to ensure comparable product yield. A simple normalization of the measured currents to the geometric surface areas (or any other determined area) ignores the geometry of the setup.

**Figure 2:**
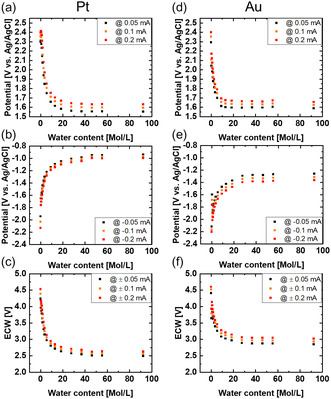
Limiting potentials extracted from the 4th cycle of each added water content (Figure 1) at 0.05, 0.1, and 0.2 mA and the corresponding ECW for a–c) platinum and d–f) gold.

For the oxidative limits (**Figure** **2**a,d), the cathodic scan direction was used and for the reductive limit (Figure 2b,e) the anodic one. The scan directions were chosen to avoid the influence of the aforementioned additional redox reactions as much as possible. Gold (Figure 2f) has a larger ECW than platinum (Figure 2c), which was mainly caused by a stronger decrease in the reductive limit potential for platinum. The oxidative limit on the other hand seemed to be independent of the electrode material. The ECW decreased by a power law in both cases and seemed to converge at ≈2.9 V (Au) and 2.6 V (Pt). The power law behavior of the ECW contrasts with the previous reported trend where a nearly linear decrease with increasing humidity in the purging gas was observed.^[^ ^18^ ^]^ However, humidity of the purging gas does not reflect how much water is taken up by the system which most likely depends on the hydrophilicity of the IL.

The large ECW at high water content is rather surprising, because the IL is then a 2.7 M aqueous salt solution and not water in IL anymore. The ECW in the presence of IL was much higher as for example in an aqueous 0.1 M KOH solution (Figure S8, Supporting Information). This may either indicate that the solvation shell of the IL is still not fully occupied, and therefore, the water molecules still did not arrange in a typically bulk water network and bond to the ions instead. A similar effect was observed by adding a salt to a humid ionic liquid where the salt binds to the water and reduces the electrosorption of water on the electrode.^[^ ^12^ ^]^ This would hinder adsorption on the electrode surface. The other suggestion would be that the IL ions accumulate at the electrode surface as shown in simulations and experiments on other IL‐charged electrode interfaces and block the surface of the electrodes.^[^ ^11^ ^]^ Simulations further suggest that water or IL molecules accumulate on the surface depending on the hydrophilicity of the IL and the electrode material.^[^ ^11^ ^]^ If the electrode is blocked by the IL, the catalytic sites of the metal would be blocked and electron would need to be transported or tunnel over the blocking IL, hence the need for higher potential. This could explain why gold and platinum had a similar oxidative limit. A third possible explanation could be that the observed potential limit is not the water oxidation but the decomposition of the organic salt destabilized by solvation with water. To gain further insight into the limiting reaction, DEMS measurements were conducted to observe the water oxidation and reduction process, namely the production of H_2_ and O_2_.

### 2.3 DEMS Measurements

**Figure 3:**
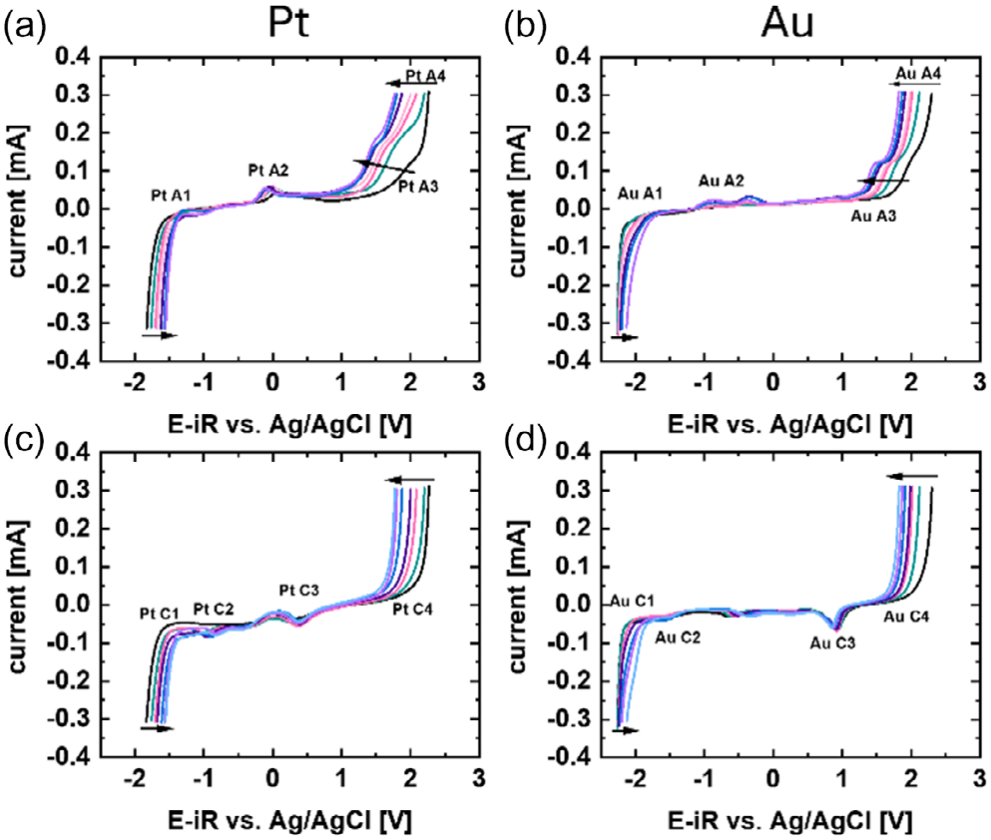
a,b) Anodic and c,d) cathodic scan direction of the 4th cycle of the CV investigations on a a,c) platinum and b,d) gold wire in the DEMS cell from 0 to 1.66 M_H2O_/L_IL_ The full water content seps and conversion to v/v% and mole‐fraction may be found in Table S2, Supporting Information.

DEMS measurements were conducted using [BMIM][OTf] with a scan rate of 100 mV s^−1^. Due to the larger starting volume, lower concentrations of water could be achieved. **Figure** **3** shows the current response for different water contents for platinum and gold electrodes. Like the experiments in a glass cell at identical sweep speed, a decrease of the ECW as well as the same redox features were observed.

The DEMS cell was outside the glovebox and a part of the assembly needed to be done outside as well so that the IL could absorb some water from the atmosphere. The duration of contact with air was minimized as much as possible and the beaker was purged with dry argon after the assembly. Coulometric Karl Fischer titration estimated a water content of ≈32 ppm (≈0.0023 M_H2O_/L_IL_) after assembly and before starting purging gas. However, some water could be taken up while the system was being purged and while the IL was first pulled through the cell. The gold reduction peak during the first cycle suggested the presence of a small amount of water.

**Figure 4:**
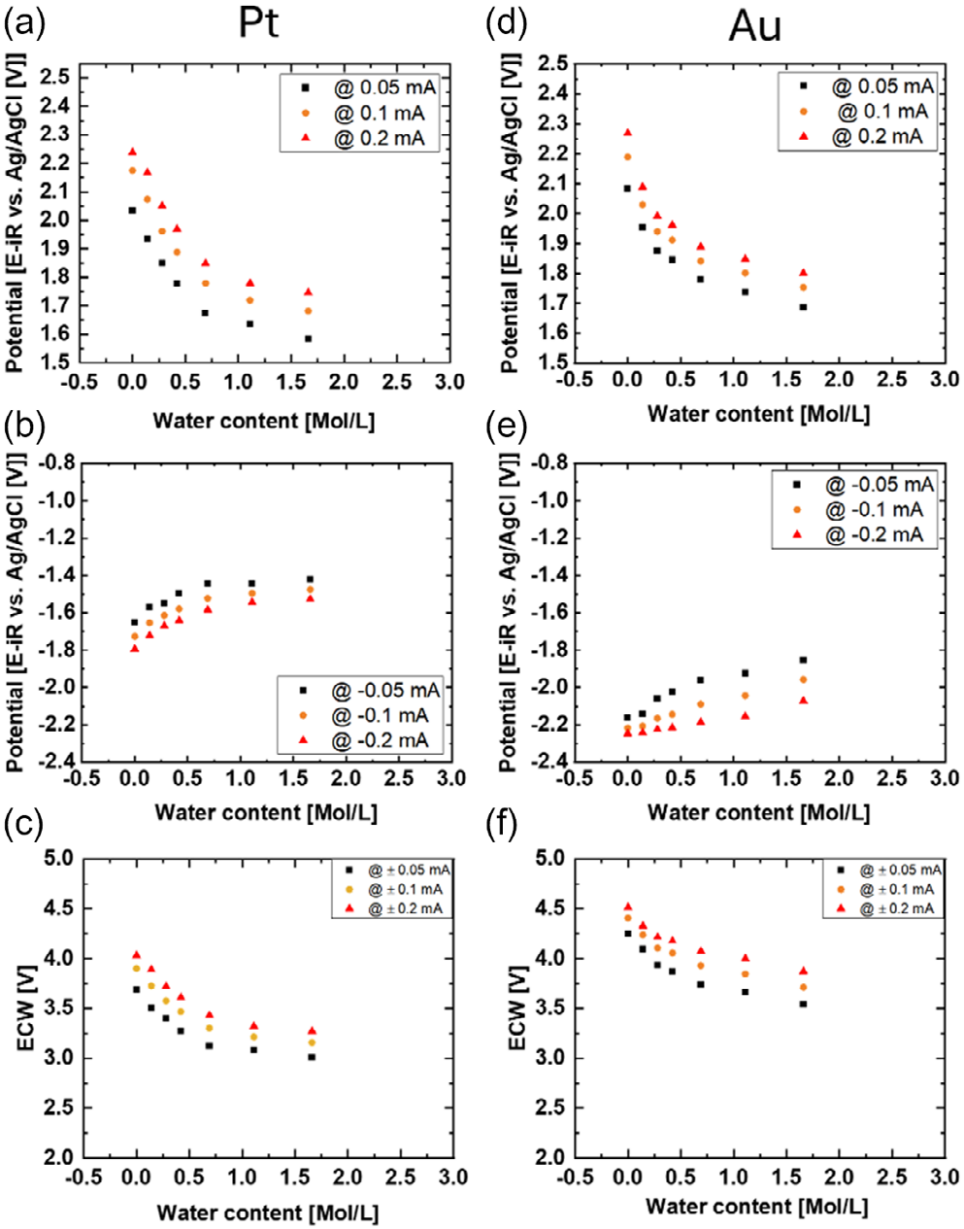
Limiting potentials extracted from the 4th cycle of each added water content (Figure 3) at 0.05 mA, 0.1 mA, 0.2 mA and the corresponding ECW for a–c) platinum and d–f) gold.

The extracted limits of the ECW are presented in **Figure** **4**. In general, a power law decrease in the ECW can be observed in both electrode materials in the DEMS flow cell, similar to the stagnant glass cell. Gold had a larger ECW as compared to platinum, which was likewise similar to the findings from the glass cell. Thus, the trends in each setup were qualitatively similar.

The CV was repeated with 10 mV s^−1^ scan rate to avoid the overlapping of signals due to the time delay between production and detection of the corresponding mass‐charge fractions. The corresponding currents and DEMS signals of [BMIM][OTf] with different water contents for the CVs on platinum and gold plates are shown in Figure S9 and S10, Supporting Information. For platinum, the reductive limit was caused by hydrogen evolution, while on gold the reduction of the cation seemed to be the limiting reaction and hydrogen was not detected for any of the investigated water concentrations (up to 1.11 M_H2O_/L_IL_).

For the limiting reaction on the anodic side, platinum had a transition from the oxidation of the triflate anion to oxygen and carbon dioxide between 0 M_H2O_/L_IL_ and 0.69 M_H2O_/L_IL_. This transition was more rapid on the gold surface, where the first water addition nearly completely changed the reaction. The time difference between the maximum potential and maximum detection peak varied with the observed species probably due to differing diffusion rates and was between 6 and 15 s which gives an estimation of the travel time in the DEMS setup and caused a clear shift between the electrochemical and mass spectroscopy data, also for 10 mV s^−1^. Therefore, the onset potential of the reaction could not be determined.

**Figure 5:**
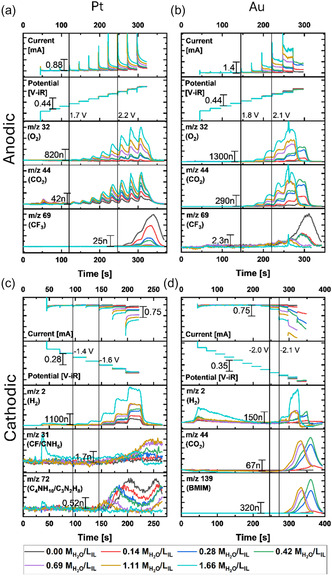
Chronoamperometry measurements of the a,b) anodic and c,d) cathodic reaction for (a,c) platinum and (b,d) gold. For additionally tracked mass‐charge ratios, see Figure S11 and S12, Supporting Information.

Due to the significant time delay between production and detection as well as product‐dependent time delays, we focused our analysis on chronoamperometry measurement coupled to mass spectroscopy (MS) as shown in **Figure** **5**, S9, and S10, Supporting Information. Key products are assigned in Table S3, Supporting Information.

The ratio *m*/*z* of 32 was assigned to O_2_ and its yield increased with the addition of water for both electrode materials for anodic potentials, suggesting oxygen evolution reaction (OER) by water oxidation (Figure 5a,b). The oxygen signal rose above the detection limit at ≈1.7 V versus Ag/AgCl. The ratio *m*/*z* of 44 was assigned to CO_2_. It was also detectable for the dried IL and likewise started rising at 1.7 V versus Ag/AgCl and with increasing water content. For platinum, a large current peak arose at the beginning of each CA step that then droped slower and nearly reached a current plateau. This profile is often attributed to charging of the electrode in the IL due to the formation of a double layer. However, the product detection signal for oxygen and carbon dioxide followed the same trend indicating that adsorbed water on or close to the surface reacted fast at each step until the concentration reached a new semi‐equilibrium. Additionally, the surface was stripped of small amounts of carbon dioxide as was also seen in the first step of the CA, where the oxygen signal did not change, but the carbon dioxide had a small signal.

Interestingly, the shape of the current profile and the shape of the product detection differed between gold and platinum. On the gold electrode, the initial spike was much less pronounced both in the electrochemical and MS data and the observed plateaus were higher in these data. Additionally, the onset of the oxygen evolution was 1.8 V versus Ag/AgCl, that is, 100 mV above that of platinum but more oxygen and carbon dioxide were detected for the same increment of water content.

The mass‐charge ratios of possible fragments of the IL anion, namely 48 (SO), 50 (CF_2_), 64 (SO_2_), 69 (CF_3_), and 99 (SO_3_F), were also tracked. Their signals were observed at high potential and decreased with increasing water content (Figure S9 and S10, Supporting Information). These signals were only observed until a water content of ≈1.11 M_H2O_/L_IL_ indicating a transition of the dominant reaction from anion decomposition to the OER. Therefore, any shift of the limiting potential defining the ECW above this threshold is related to a change in the kinetics of the OER. The onset of the decomposition did not change with water content as also previously described in literature.^[^ ^13^ ^]^ The CF_3_ signal (*m*/*z* 69) was selected to represent the trends of IL decomposition in Figure 5. Its signal was lower on the gold surface and decreased faster as compared to the platinum surface, which indicated in summary that OER electrocatalysis was faster on gold than on platinum in BMIM Triflate.

For cathodic potentials, hydrogen was clearly seen on the platinum electrode for all potentials and water concentrations, again suggesting water reduction as the source (Figure 5c). Additionally, the mass‐charge ratios of 31 (CF or CNH_5_) and 72 (C_4_H_10_N) were detected as decomposition products of the anion and cation, respectively. The C_4_H_10_N signal decreased with increasing water content suggesting a transition from cation decomposition to HER, also at a water threshold of 1.11 M_H2O_/L_IL_. Interestingly, both signals rose at open‐circuit after the most cathodic potential, which suggests that a reactive species was produced that decomposed both the anion and cation. A detailed study of this observation is beyond the scope of this work. In summary, it remained unclear if the hydrogen originated exclusively from water in the electrolyte or if some might originate from the cation. However, the hydrogen signal strongly increased with the water content supporting water as the main source.

The gold electrode showed a different behavior for cathodic potentials. The hydrogen detection rose above the baseline only above −2.0 V versus Ag/AgCl for high water content. Several other masses, including 44 (CO_2_ or C_2_H_6_N) and 139 (BMIM), were detected to increase in the same potential range but only for intermediate water content. The detection of multiple different mass‐charge ratios in Figure S12, Supporting Information (see Table S3, Supporting Information, for possible fragments), indicated a decomposition of the cation. We draw the following conclusions for gold from these observations: 1) water can increase the IL decomposition; 2) cation decomposition is strongest at intermediate water content; 3) hydrogen evolution required high water content. It became the dominant reaction at 1.66 M_H2O_/L_IL_ showing a sharp transition with water content as opposed to the more gradual changes discussed previously.

We advise to only analyze the intensity of the MS signals qualitatively, due to a possible distortion of the signal strength due to increasing amount of water vapor in the vacuum with increasing water content in the IL (Figure S13, Supporting Information).

At the beginning of the cathodic potential steps on both platinum and gold, we observed a small drop in the oxygen signal (Figure S11 and S12, Supporting Information; O_2_ signal after 45 s, where the polarization starts). Due to the background variation in the oxygen signal, it was not possible to quantify the amount of oxygen, but it was observed for all water contents indicating that some residual oxygen is present in the electrolyte even after purging with argon. This further supported our hypothesis that the Pt C3 and Au C2 peak in Figure 1 and 3 originate from the ORR.

Due to the very similar trend in specific groups of mass‐charge ratios (e.g., *m*/*z* 139, 125, 44 etc. and 69, 64, 50, etc.), it was not clear if the electrochemical decomposition created the observed fragments or if they are a product of fragmentation during ionization in the mass spectrometer. In the case of *m*/*z* = 139 (BMIM), a charge neutralization reaction of the cation to a neutral molecule and then fragmentation during ionization is a plausible explanation. This might also be the case for the triflate anion (*m*/*z* = 149). To investigate this, further chronopotentiometry measurements were conducted (Figure S14, Supporting Information) on the anodic and cathodic side of the ECW for constant product production. *m*/*z* = 139 (BMIM) was detected; however, *m*/*z* = 149 (Triflate) could not be detected. This indicates that triflate decomposes in the electrolyte. For BMIM, at least some fraction seemed to neutralize during the reduction and then was ironized in the mass spectrometer.

The water content of the dry IL needs further discussion. After assembly, 32 ppm (≈0.0023 M_H2O_/L_IL_) was measured by KF titration; however, residual humidity in the cell and in the argon purging gas (<2 ppm) probably increased the water content slightly after assembly. [BMIM] [OTf] takes up water quite rapidly. When the IL was left exposed for 15 min to air in the fume hood under air flow, 2294 ppm (0.166 M_H2O_/L_IL_) were measured. Assuming linearity, the uptake was estimated as about 151 ppm (≈0.011 M_H2O_/L_IL_) per minute. The typical handling time in air was 1 min or less, giving a background of around 183 ppm (0.013 M_H2O_/L_IL_) for nominally water‐free IL, which is an order of magnitude below the lowest addition of 0.14 M_H2O_/L_IL_. We note that an increment of 0.14 M_H2O_/L_IL_ water had a strong effect on the anodic oxygen and carbon dioxide detection for gold and platinum. If the original water content was 0.14 M_H2O_/L_IL_ or higher, then we could detect oxygen, which was not the case. The nearly complete suppression of the anodic IL decomposition on the gold surface for the lowest water contents further supported an adventitious water content well below 0.14 M_H2O_/L_IL_ of the nominally water‐free IL that agrees with our estimation from KF titration.

## 3 Conclusion

We found that the ECW of [BMIM][Otf] decreased by a power law with increasing water content on platinum and gold electrodes. The ECW of a gold electrode was larger than that of the platinum electrode, demonstrating the importance of the electrode material to understand the ECW. Our DEMS measurements showed that IL decomposition at low water content was quenched by the OER above 1.11 M_H2O_/L_IL_ where the OER by water oxidation contributed to the oxidative limit on both platinum and gold with similar onset. Moreover, gold had a 100 mV higher onset potential as compared to platinum but showed larger and more constant oxygen production. The reductive limit on platinum was associated with HER likely from water as it increased with water content. The reductive reaction on gold was assigned to the decomposition of the cation except for the highest investigated water content of 1.66 M_H2O_/L_IL_ where the reaction switched to hydrogen evolution. The independence on hydrogen evolution made the ECW of gold less sensitive to small water contents during the experimental setup. Our work highlights that both the electrolyte and the electrode material determined the reactions limiting the ECW. The assignment of the reactions required product determination. This insight is relevant for researchers investigating ILs for a variety of applications, especially ones that involve contact with a water source, such as air, to understand the possible reasons for achievable limits in their setup depending on the electrode material used.

## 4 Experimental Section

4.1

4.1.1

4.1.1.1

1‐Butyl‐3‐methylimidazolium triflate was used without further purification (except for drying as described below). A glass cell was used as an electrochemical cell (Figure S1a, Supporting Information), with a leakless Ag/AgCl electrode (eDAQ) as the reference electrode, a curled platinum wire (Götze Gold, 999) as the counter electrode, and either a platinum (Götze Gold, 999) or a gold (Götze Gold, 999) wire as working electrode. The leakless reference electrode was chosen to avoid contamination with silver from an Ag/Ag+ reference electrode while maintaining a fixed reference when changing the electrolyte. The working electrode wires were partially covered by a PTFE heat shrink tube, to ensure a constant surface area while adding water to the electrolyte.

The IL was dried at 70–80 °C in a flask with a heater jacket and ≈7 E−3 mbar in a vacuum chamber for >72 h. After drying, the chamber was flooded with pure argon (Alphagaz, 99 999%) and the flask was transferred into a glovebox with argon atmosphere (O_2_ <0.5 ppm, H_2_O <0.5 ppm). 3 mL of the IL in the glovebox was filled into the electrochemical cell and the working and counter electrodes were added. The third port was closed and the cell was transferred out of the glovebox to the electrochemical measurement station. The Ag/AgCl reference electrode was rinsed with acetone and then dried in air to remove water. Afterward, the free port was opened, and the Ag/AgCl reference electrode was added and directly closed to minimize the air contact. The cell was then connected to a Gamry 1010 potentiostat in a 3‐electrode configuration.

The electrochemical measurement included three steps for each water content and material: (i) a measurement of the open‐circuit potential, (ii) impedance spectroscopy between 2 MHz and 1 Hz, and (iii) CV with 5 cycles. Each cycle started at 0 V versus Ag/AgCl and was scanned in the anodic direction until a current of 0.3 mA was reached, and then the potential was scanned in the cathodic direction until a current of −0.3 mA was reached. Finally, the potential was again scanned in the anodic direction to 0 V versus Ag/AgCl. The next scan was started immediately. After the three steps were conducted on the platinum electrode, the potentiostat was connected to the gold electrode. The three steps were repeated immediately to ensure that the measurement on both electrodes was conducted in the same electrolyte with the same water content. Following the measurement on both electrodes, a small amount of water was added to the electrolyte in the range of 0.66% v/v (0.369$M_{H_{2}O}/L_{\text{IL}}$) to 60% v/v (92.2 $M_{H_{2}O}/L_{\text{IL}}$, ≈2.7M Il in H_2_O). The exact steps can be found in Table S1, Supporting Information.

All electrochemical CV data were iR‐compensated using the resistance extracted from electrochemical impedance measurements (Figure S2, Supporting Information). The resistance was taken as the real part of the impedance where the phase was closest to 0° between 10 and 20 kHz. The procedure is further discussed below. The potential at 0.05 mA, 0.1 mA, and 0.2 mA in the cathodic scan direction was extracted for the anodic limit of the ECW. The same procedure was done for the cathodic limit in the anodic scan at −0.05 mA (−0.1 mA and −0.2 mA). A surface normalization to compare measurements was not considered reasonable, because the geometry of the setups is different. Additionally, the distance between the different points of the wire to the counter electrode was not uniform. This affects the field lines and therefore the contribution of the different areas to the current.

For the DEMS measurements, 20 mL of dried [BMIM][OTf] was filled into a glass reservoir which was connected to an argon line. A constant stream of argon onto the surface of the IL was realized to avoid underpressure and inflow of air into the reservoir. A water content after assembly (before Ar purging) of ≈32 ppm was determined using coulometric Karl Fischer titration. The DEMS cell is of a similar design as described previously^[^ ^25^ ^]^ for the double electrode flow cell but with a membrane (cobetter filtration PF‐B002HS‐T) covered inlet between working electrodes made of platinum (Götze Gold, 999) and gold (Götze Gold, 999; Figure S1b, Supporting Information). Both electrodes consist of a polished 10 × 5 × 1 mm plate of which an area of 5 × 5 mm is exposed to the electrolyte in the channel. The reference electrode is the same leakless Ag/AgCl as used for the other experiments. The vacuum system consists of a prechamber connected to the membrane which is pumped using a scroll pump to ≈10^−2^ mbar. A pipe with an orifice, a shutter, and a pressure gauge connects the prechamber to the analysis chamber of the quadrupole mass spectrometer (MKS). During the measurements, the prechamber is pumped via the MS‐setup to avoid the backflow of air through the scroll pump and thereby reducing the oxygen background pressure. This is possible with ionic liquids in comparison to other solvents due to the low vapor pressure. However, it limits the maximum possible addition of water.

The flow through the cell is realized by pulling the IL through the cell for the platinum measurements using a syringe pump and pushing it back for the gold measurements. For each water aliquot addition, the water was added with an mL syringe into the reservoir without opening the system. After adding water, the electrolyte was shaken and some were pulled into the cell before the electrochemical measurements. The electrochemical procedure described above was repeated in the DEMS cell with added water contents between 0 V/V% water and 2.9 V/V% (1.7 $M_{H_{2}O}/L_{\text{IL}}$). The exact steps can be found in Table S2, Supporting Information. After 5 CVs with 100 mV s^−1^, a slower CV with 10 mV s^−1^ was conducted to give the product more time to enter the mass spectrometer system through the membrane and vacuum system. Additionally, after the CVs, chronoamperometry measurements with steps of increasing absolute potentials were conducted to better determine the potential of the start of the reactions. Each step was 100 mV. Fifteen mass‐charge ratios were selected for analysis based on previous experiments.

## Conflict of Interest

The authors declare no conflict of interest.

## Supporting information

Supplementary Material

## Data Availability

The data that support the findings of this study are openly available in Zenodo at [https://doi.org/10.5281/zenodo.17292403], reference number [17292403].
