# Supplementary material for: Different Reactions Define the Electrochemical Window in 1‐Butyl‐3‐Methylimidazolium Triflate on Gold and Platinum Electrodes
Source: Chemphyschem. 2025 Oct 16;26(23):e202500096. doi: 10.1002/cphc.202500096 (PMC12677712; doi:10.1002/cphc.202500096)
Supplement: Supplementary file 1 — Supplementary Material [file CPHC-26-e202500096-s001.pdf]

Supporting Information

Different Reactions Define the Electrochemical Window in 1-Butyl-3-Methylimidazolium Triflate on Gold and Platinum Electrodes

Frederik J. Stender[a], Marcel Risch[a,b]*

[a] Frederik Johannes Stender, Dr. Marcel Risch
Institut für Materialphysik
Georg-August-Universität Göttingen
Friedrich-Hund-Platz 1, 37085 Göttingen
[b] Dr. Marcel Risch
Nachwuchsgruppe Gestaltung des Sauerstoffentwicklungsmechanismus
Helmholtz-Zentrum Berlin für Materialien und Energie GmbH
Hahn-Meitner-Platz 1, 14109 Berlin
E-mail: marcel.risch@helmholtz-berlin.de

Table of Contents

Supporting Figures
Figure S1. Used setups
Figure S2. Electrochemical impedance spectroscopy glass cell
Figure S3. Extracted real resistance for the glass cell
Figure S4. Electrochemical impedance spectroscopy DEMS
Figure S5. Extracted real resistance for the DEMS cell
Figure S6. Electrochemical impedance spectroscopy internal resistance
Figure S7. Influence of oxygen and argon saturation
Figure S8. Comparison between IL and 0.1 M KOH
Figure S9. DEMS signal response to CV measurements on platinum
Figure S10. DEMS signal response to CV measurements on gold
Figure S11. DEMS signal response to CA measurements on platinum
Figure S12. DEMS signal response to CA measurements on gold
Figure S13. Effect of added water on H_2 baseline
Figure S14. CP measurements investigating the BMIM and OTf molecules

Supporting Tables
Table S1. Water addition for glass cell measurements
Table S2. Water addition for DEMS measurements
Table S3. Possible fragments for observed mass-charge-ratios

Supporting Figures

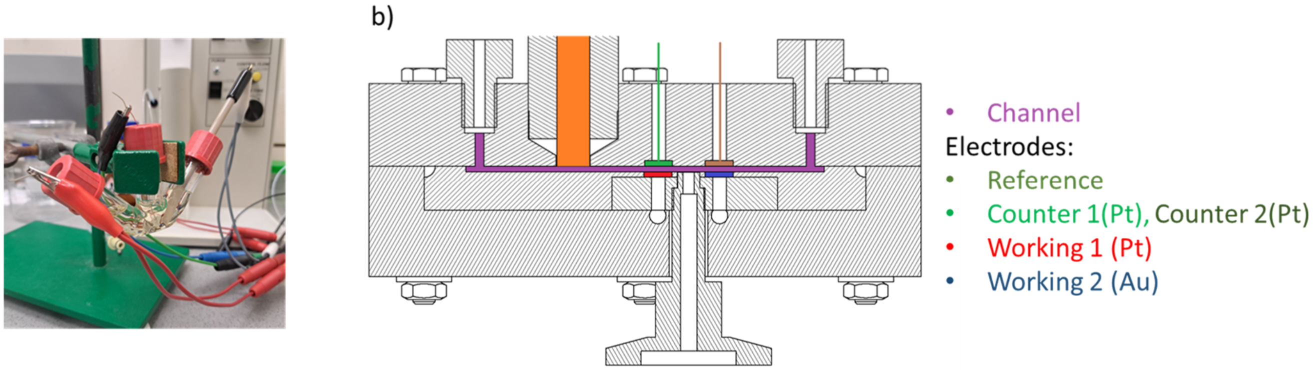

Figure S1: *Used (a) 3-electode glass cell and (b) DEMS setup.*

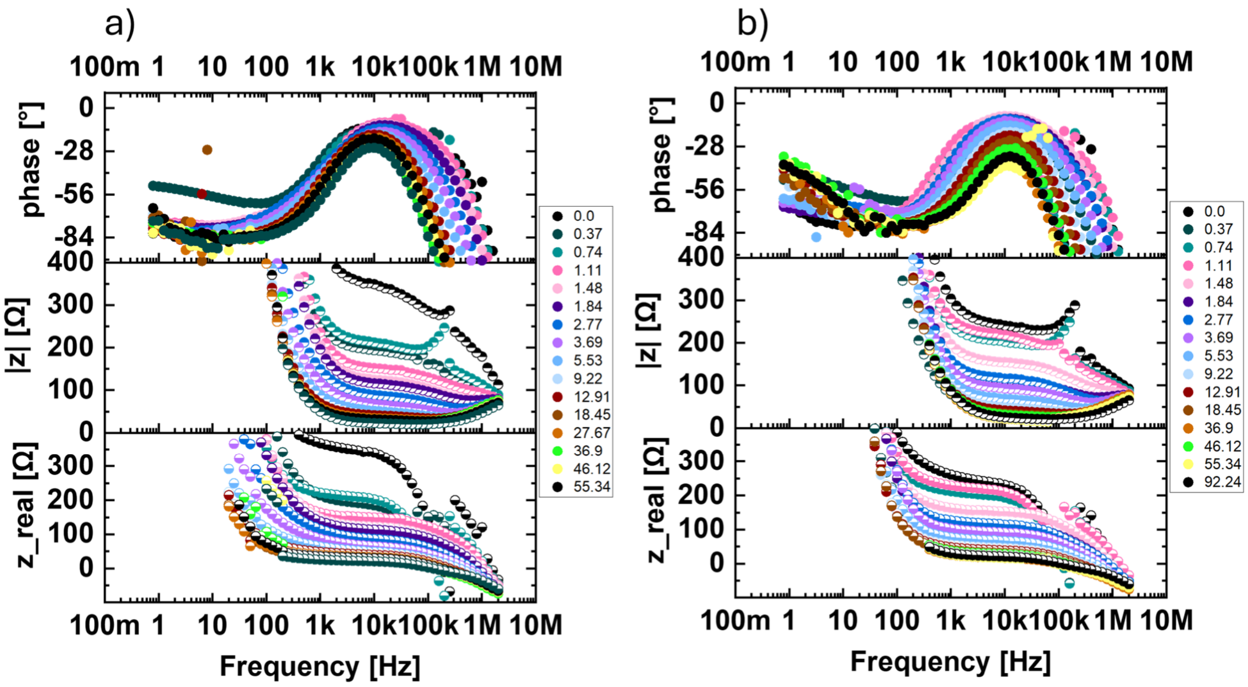

Figure S2: *Measured EIS spectra for (a) platinum and (b) gold wire with different water contents in the 3-electrode glass cell*

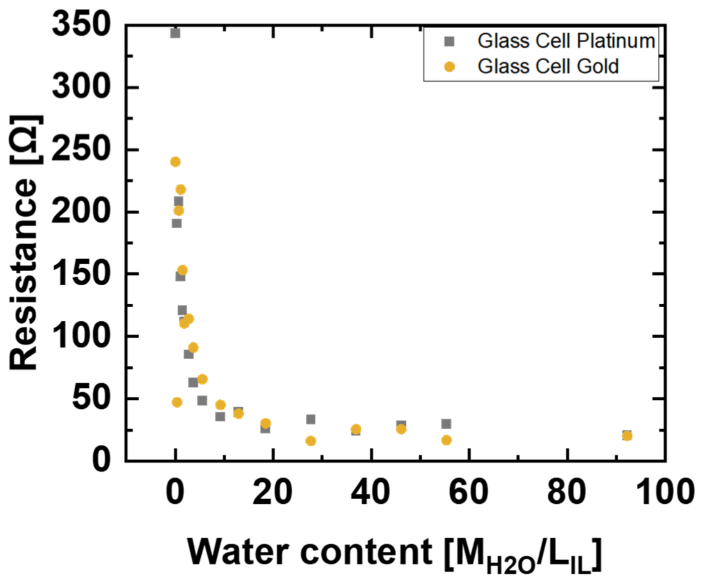

Figure S3: *Extracted resistance for the Ionic liquids with different water contents in the 3-electrode glass cell.*

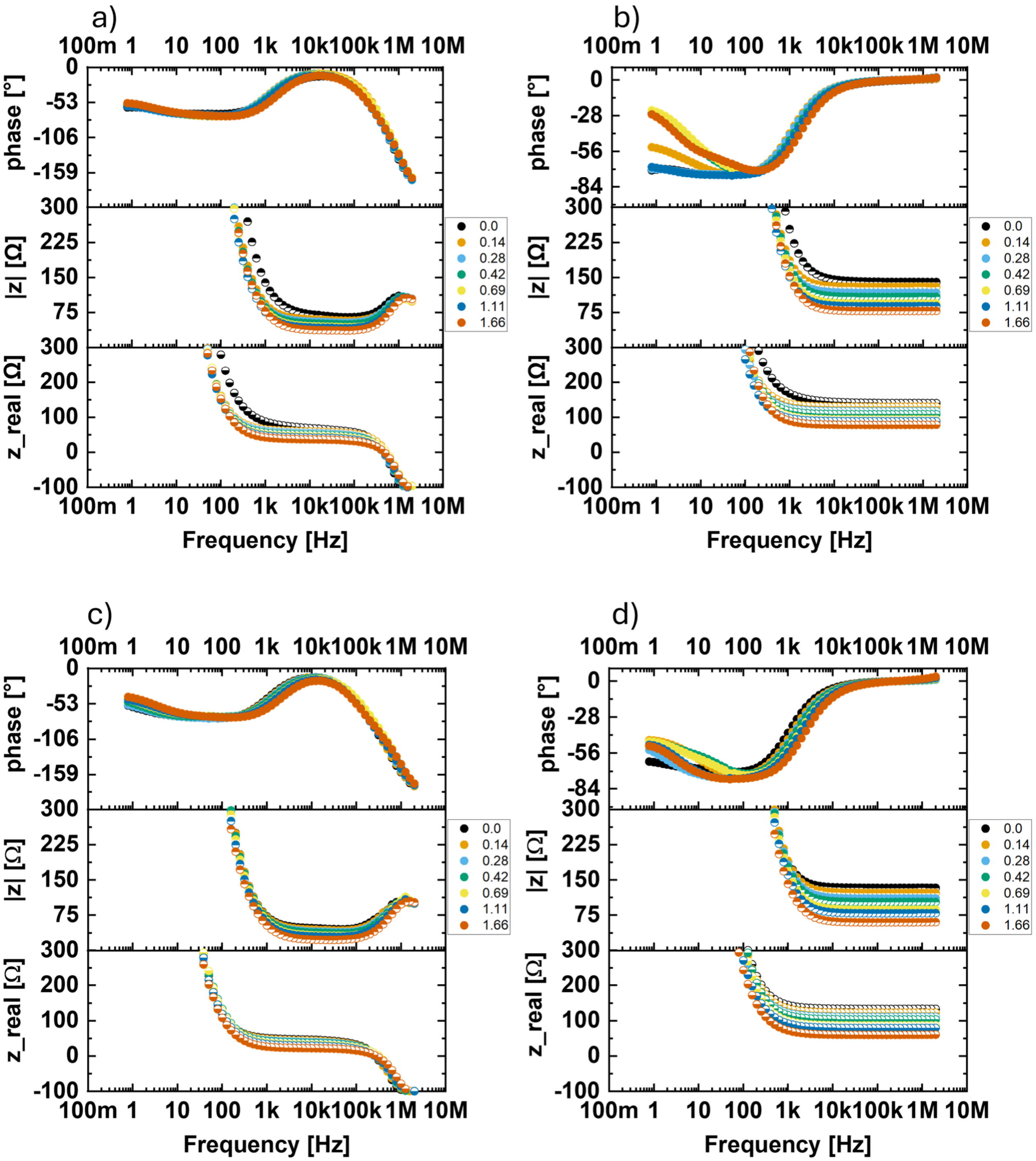

Figure S4: *(a,c) 3-electrode and (b,d) 2-electrode EIS measurements in the DEMS cell for (a,b) platinum and (c,d) gold. Concentrations in* M_{H2O}/L_{IL}.

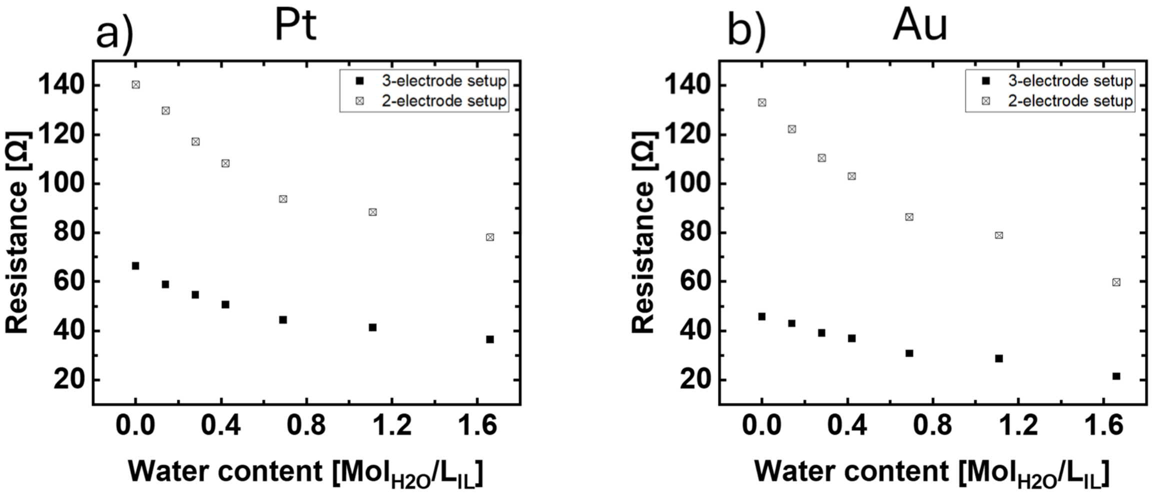

Figure S5: *The extracted resistances for (a) platinum and (b) gold show a similar trend but lower real resistances for the 3-electrode setup.*

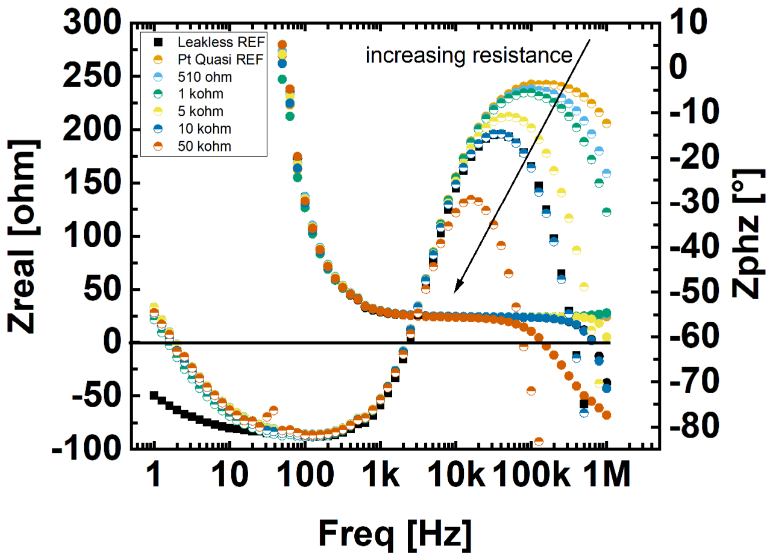

Figure S6: *Test of the effect of the artefact of the leakless reference electrode on the measured real resistance. Measurements were made in the glass cell filled with HMIM Triflate with ~40 v/v % H2O. A platinum wire was inserted at the same position as the reference electrode and used as a quasi-reference electrode. Resistors were then added to the platinum wire and EIS spectra measured. It is shown here clearly that the effect of a higher resistance in the reference electrode leads to the artefact of negative real resistance at high frequences. However, the extracted real resistance at phase closest to 0 is not significantly affected due to the plateau in the real resistance part. The leakless reference electrode behaves highly like the Pt quasi-reference-electrode with an added 10 kOhm resistor.*

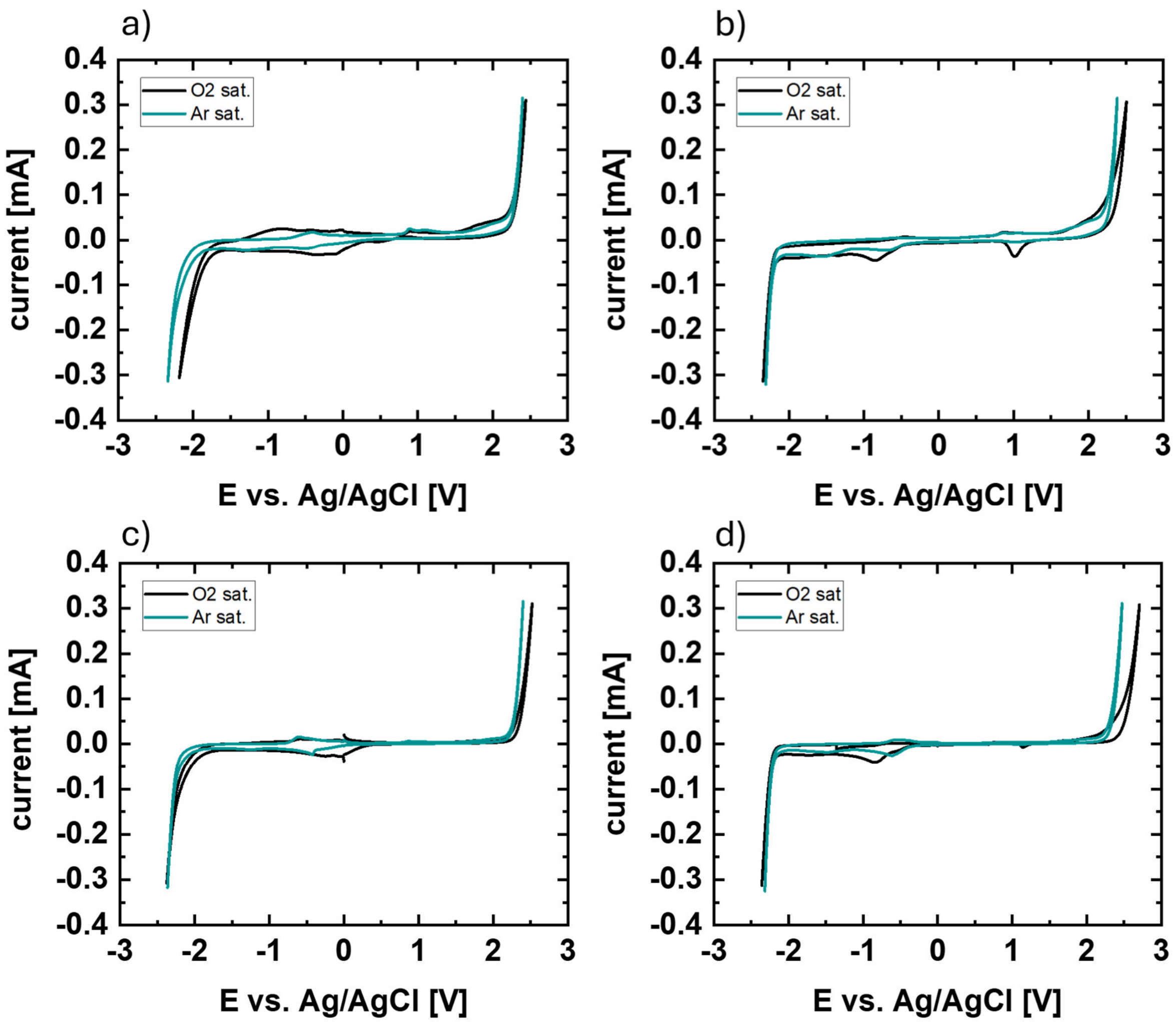

Figure S7: *Influence of argon and oxygen saturation on the electrochemical behavior of BMIM Triflate on (a,c) platinum and (b,d) gold with (a,b) 100mV/s and (c,d) 10 mV/s scan rates.*

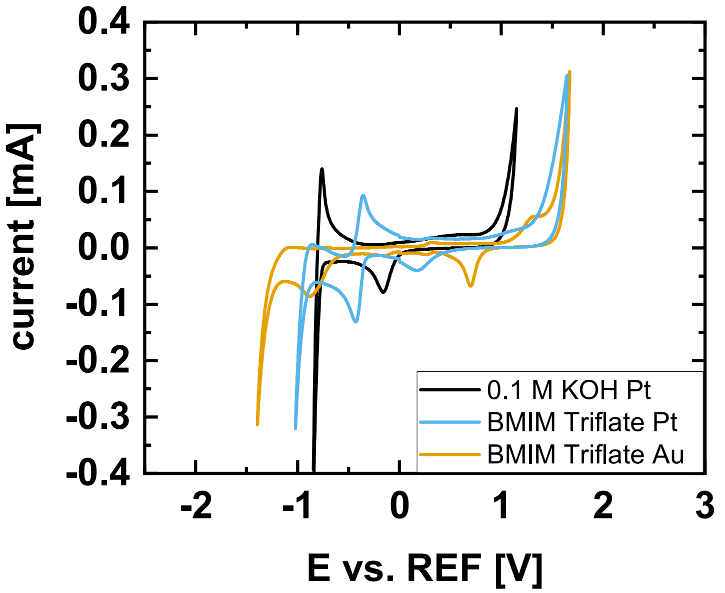

Figure S8: *Comparison of the 4th cycle between ionic liquid with the highest concentration of water (92 M_{H2O}/L_{IL}) on gold and platinum and 0.1 M KOH.*

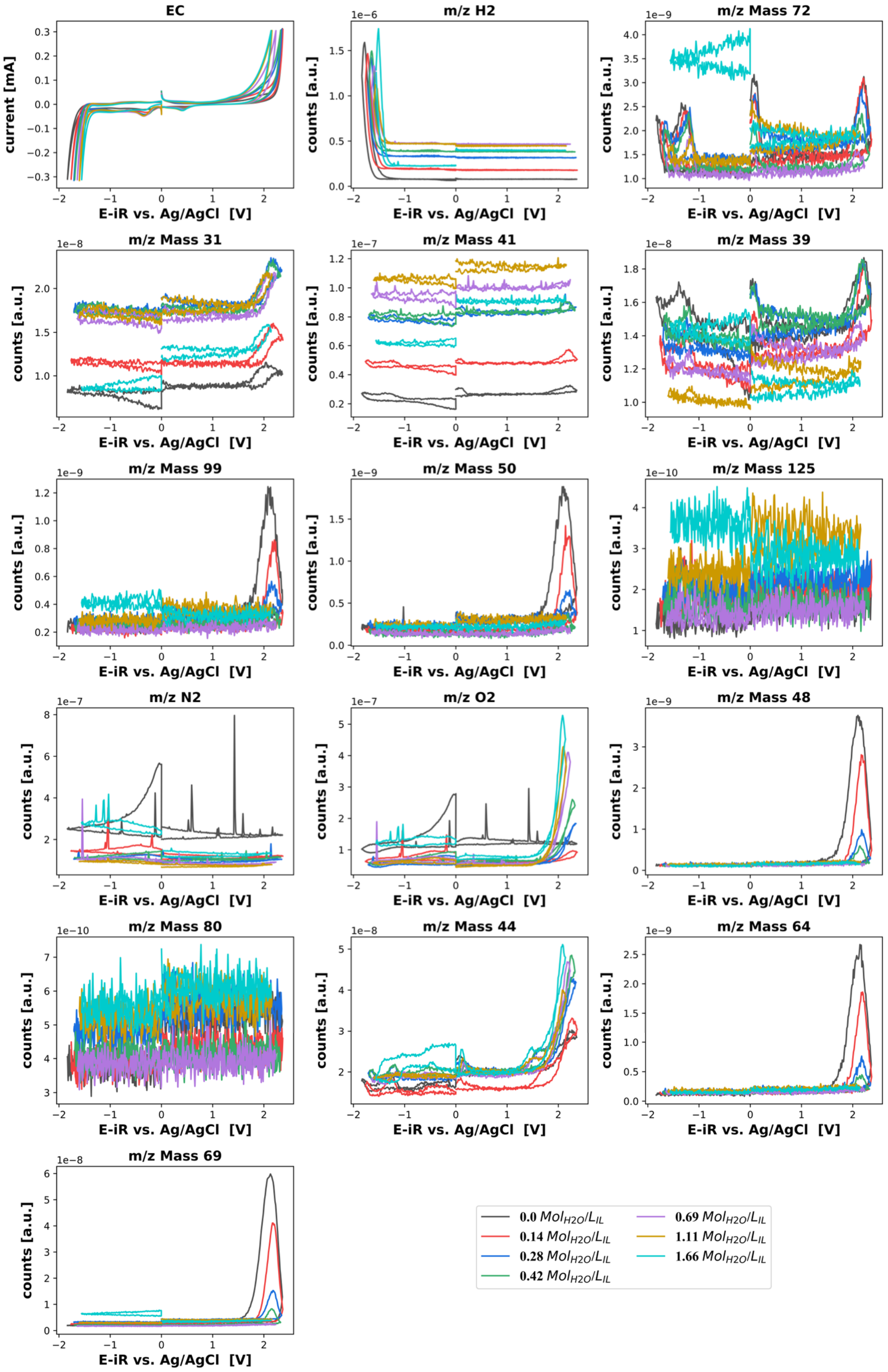

Figure S9: *DEMS responses for the 15 observed mass to charge ratios investigated during the CV on platinum with 10 mV/s scan rate. The background was not subtracted explaining the different heights and jumps between the anodic and cathodic side which were performed with a small break in between. The increase of the background hydrogen signal indicates that the water content in the RTIL increases, as hydrogen is a fragment product of water during ionization in the mass spectrometer.*

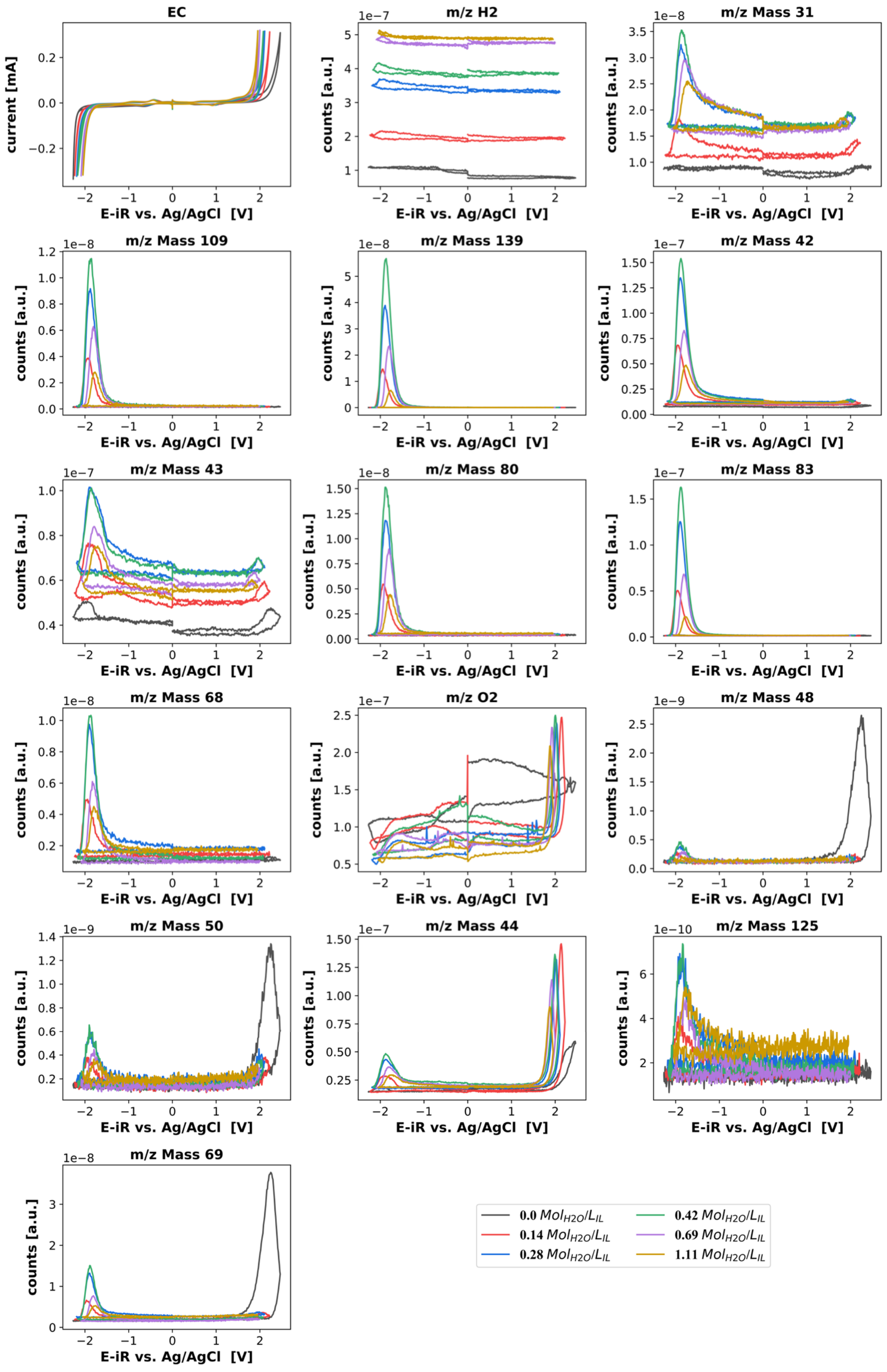

Figure S10: *DEMS responses for the 15 observed mass to charge ratios investigated during the CV on gold with 10 mV/s scan rate. The background was not subtracted explaining the different heights and jumps between the anodic and cathodic side which were performed with a small break in between. The increase of the background hydrogen signal indicates that the water content in the RTIL increases, as hydrogen is a fragment product of water during ionization in the mass spectrometer.*

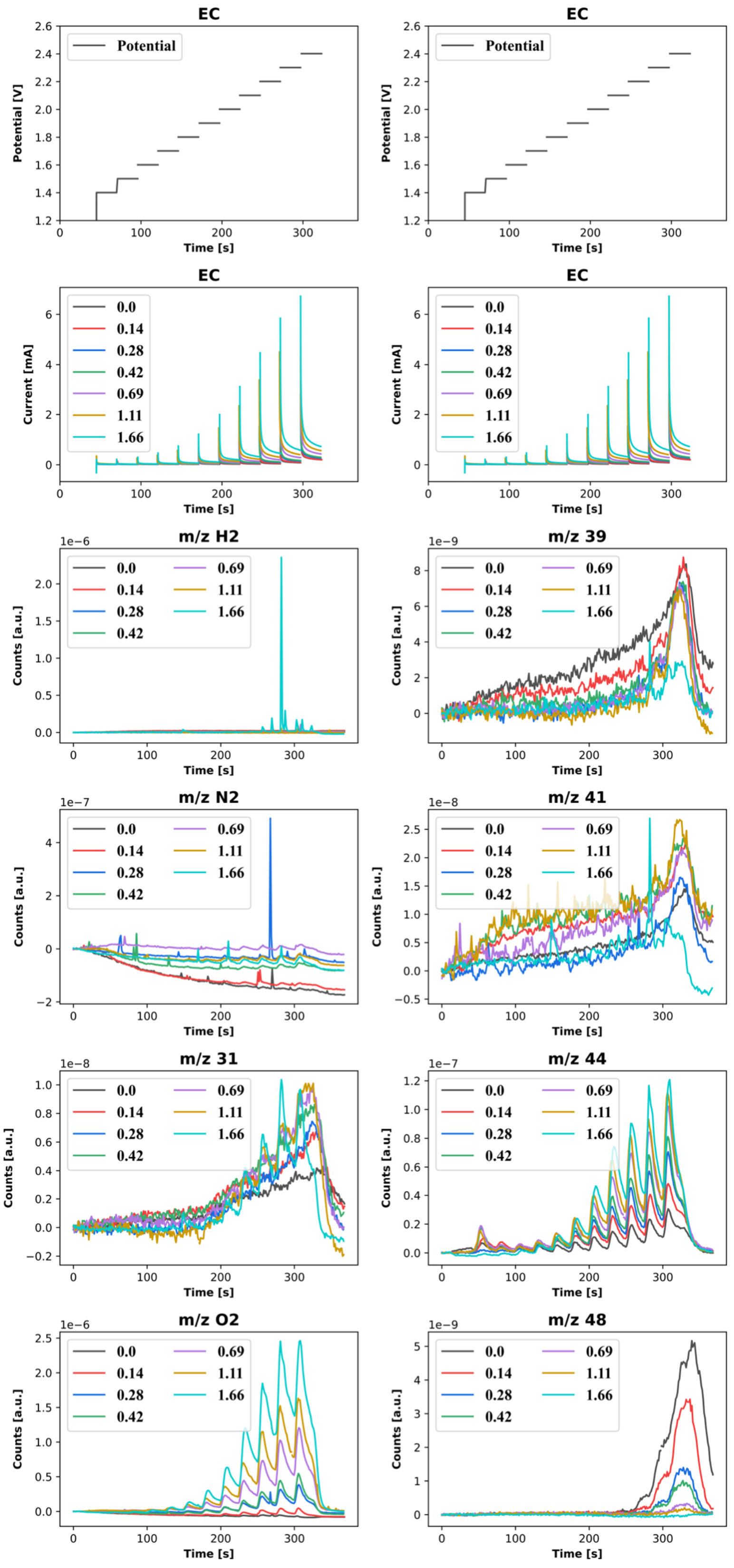

Figure S11a: *Observed mass charge ratios from 1 to 48 for the CA measurements on platinum for the anodic potentials. Background was subtracted.*

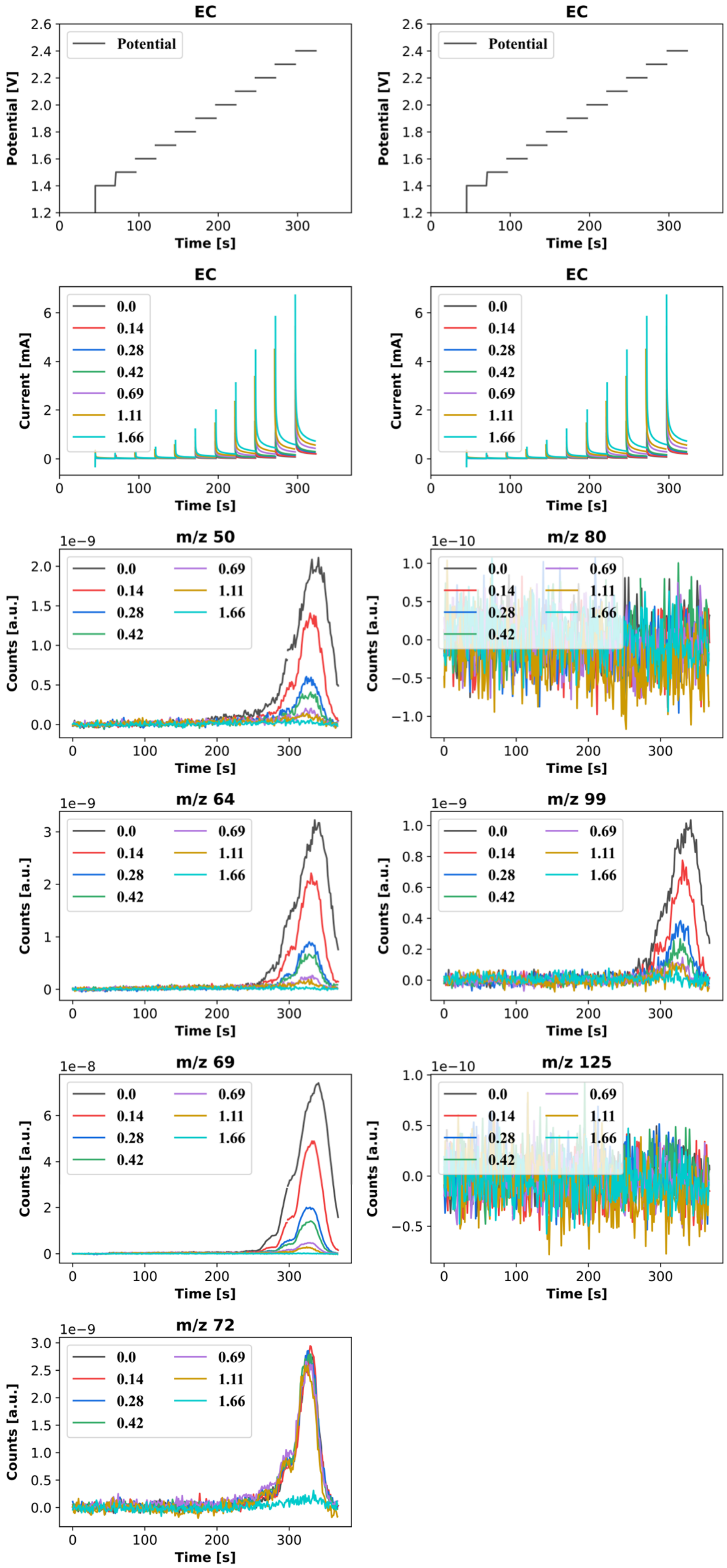

Figure S113b: *Observed mass charge ratios from 50 to 125 for the CA measurements on platinum for the anodic potentials. Background was subtracted.*

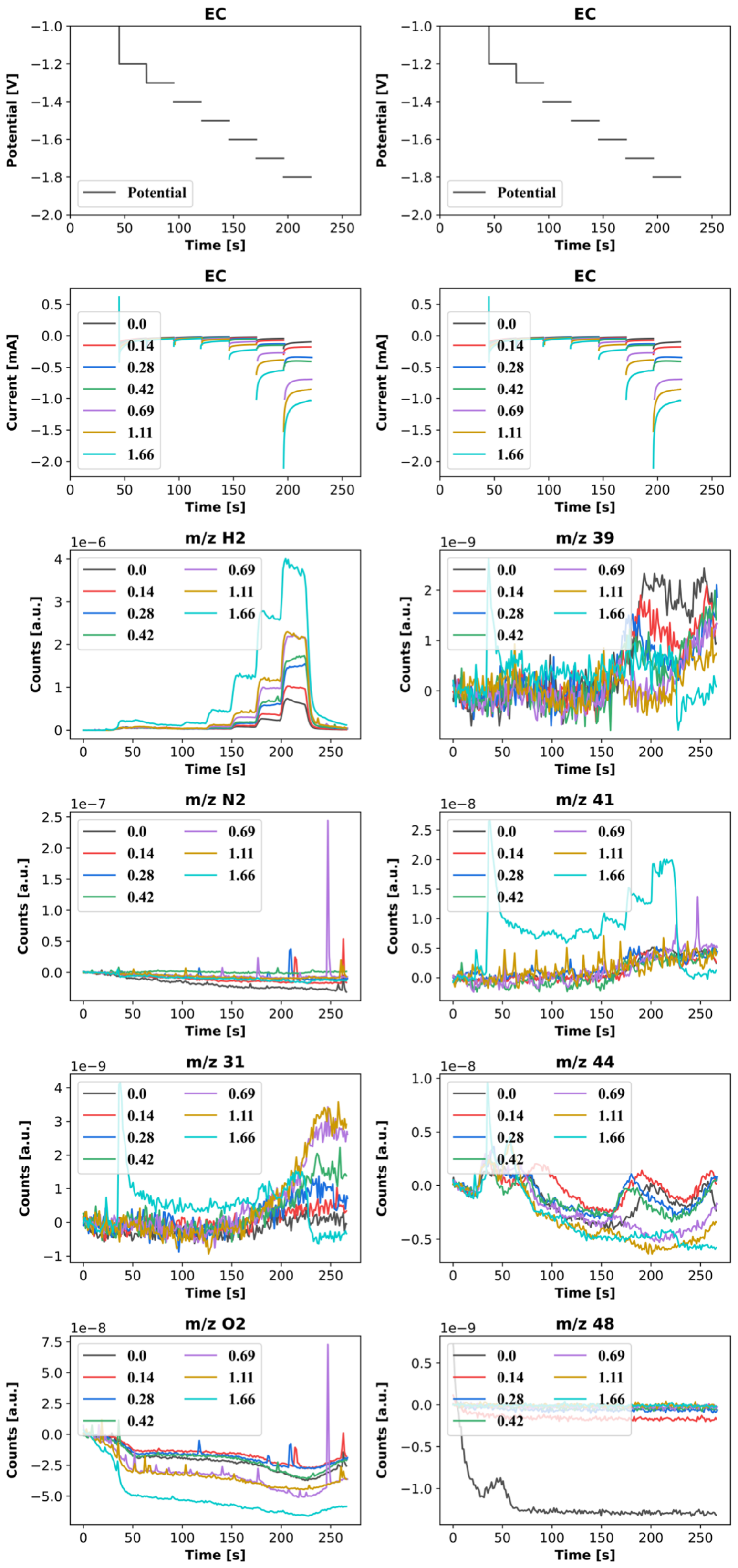

Figure S11c: *Observed mass charge ratios from 1 to 48 for the CA measurements on platinum for the cathodic potentials. Background was subtracted.*

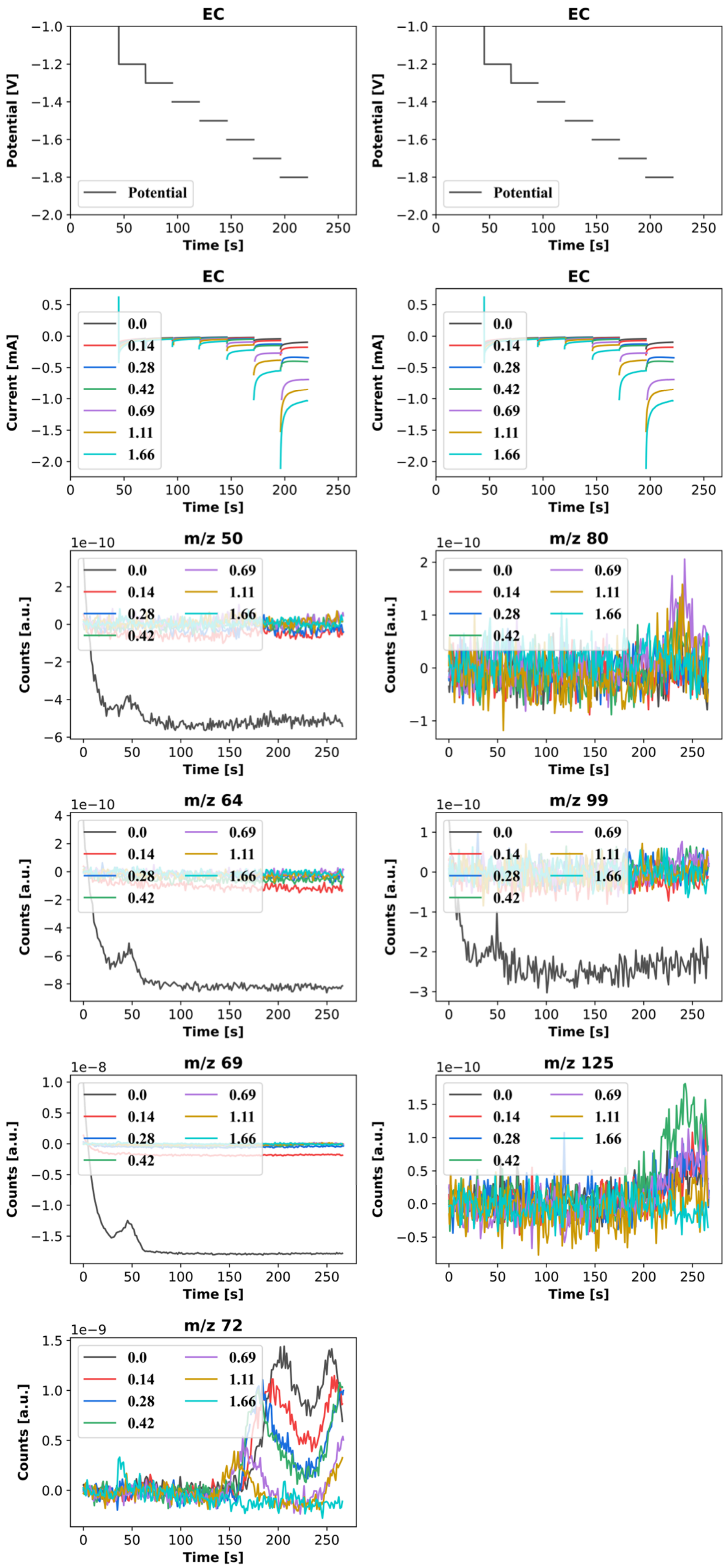

Figure S11d: *Observed mass charge ratios from 50 to 125 for the CA measurements on platinum for the cathodic potentials. Background was subtracted.*

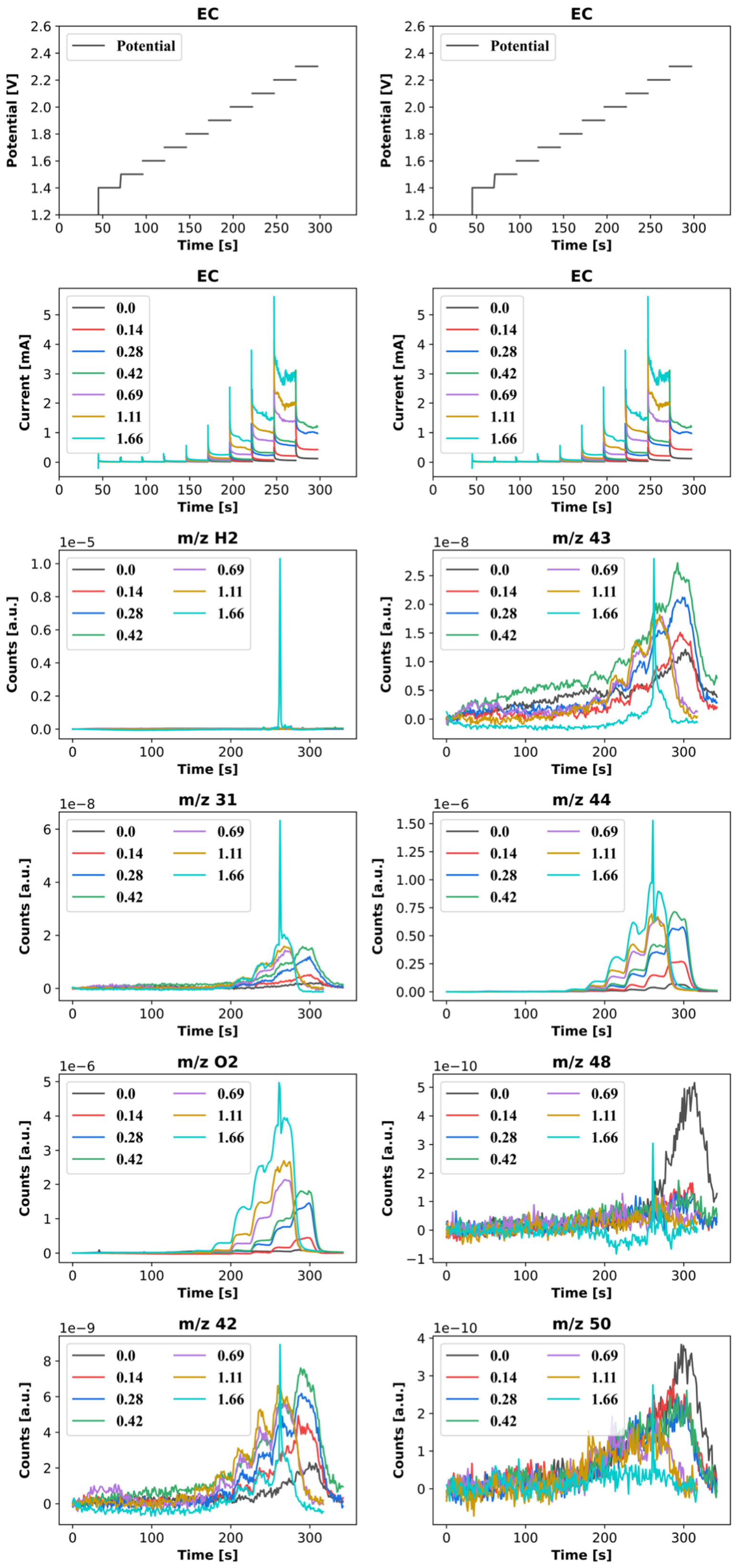

Figure S124a: *Observed mass charge ratios from 0 to 50 for the CA measurements on gold for the anodic potentials. Background was subtracted.*

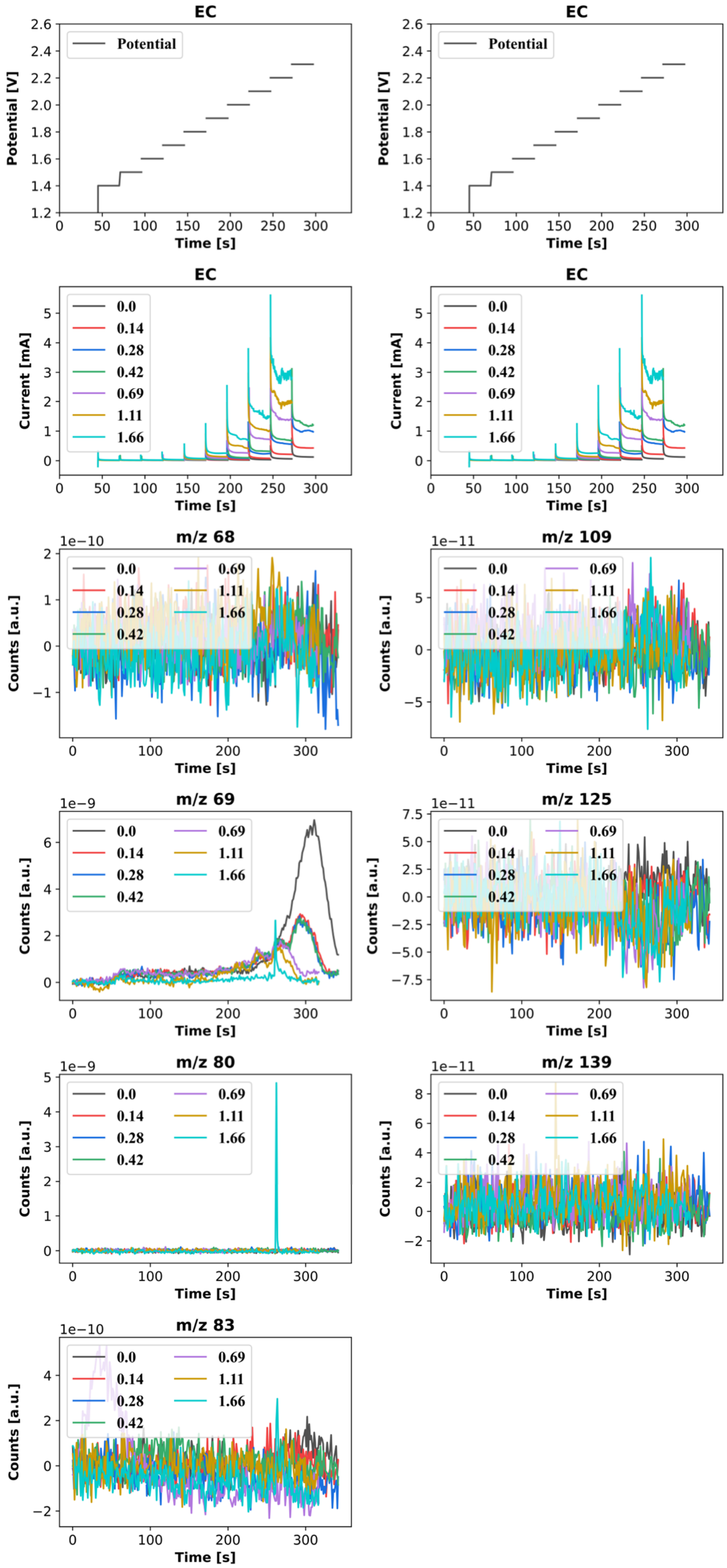

Figure S12b: *Observed mass charge ratios from 68 to 139 for the CA measurements on gold for the anodic potentials. Background was subtracted.*

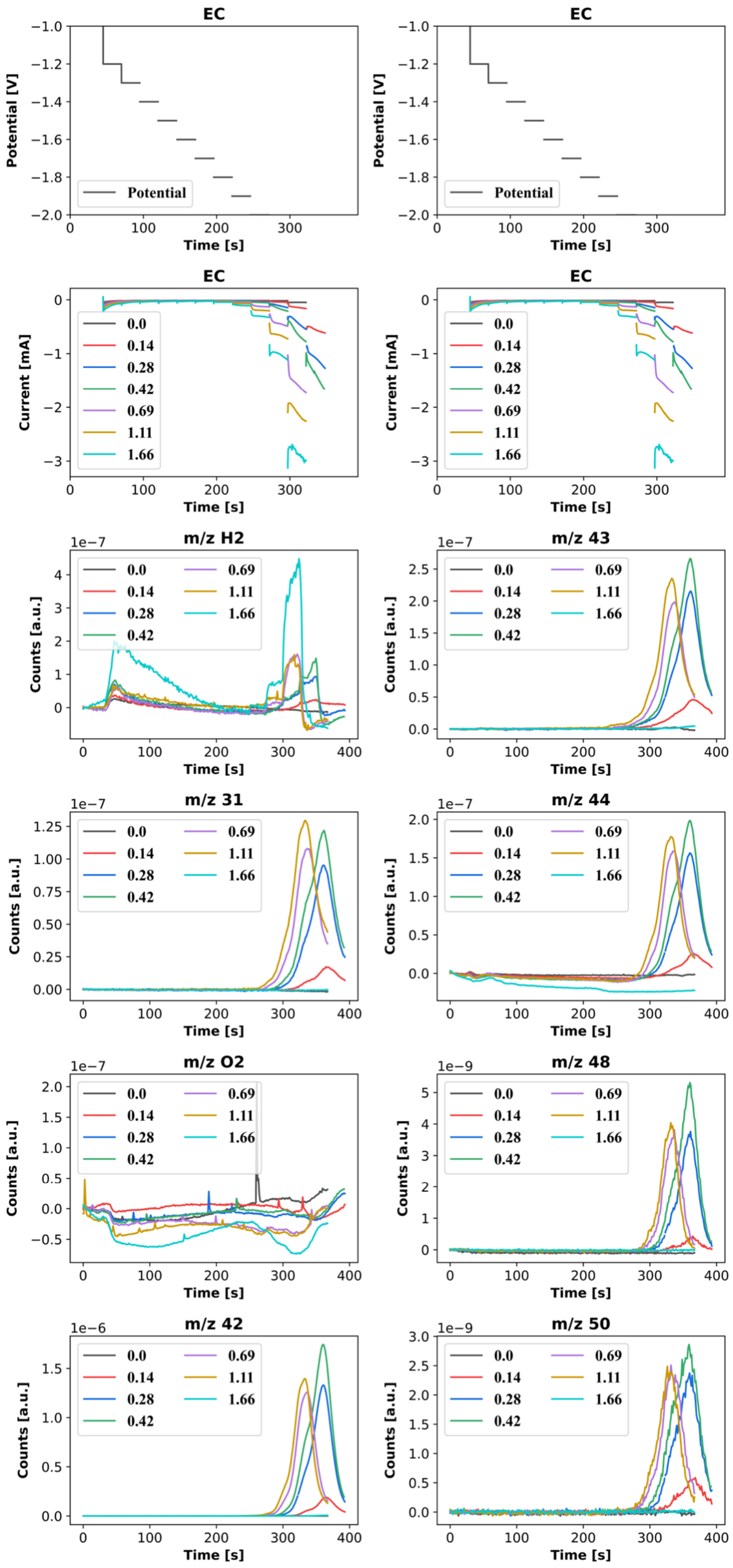

Figure S12c: *Observed mass charge ratios from 0 to 50 for the CA measurements on gold for the cathodic potentials. Background was subtracted.*

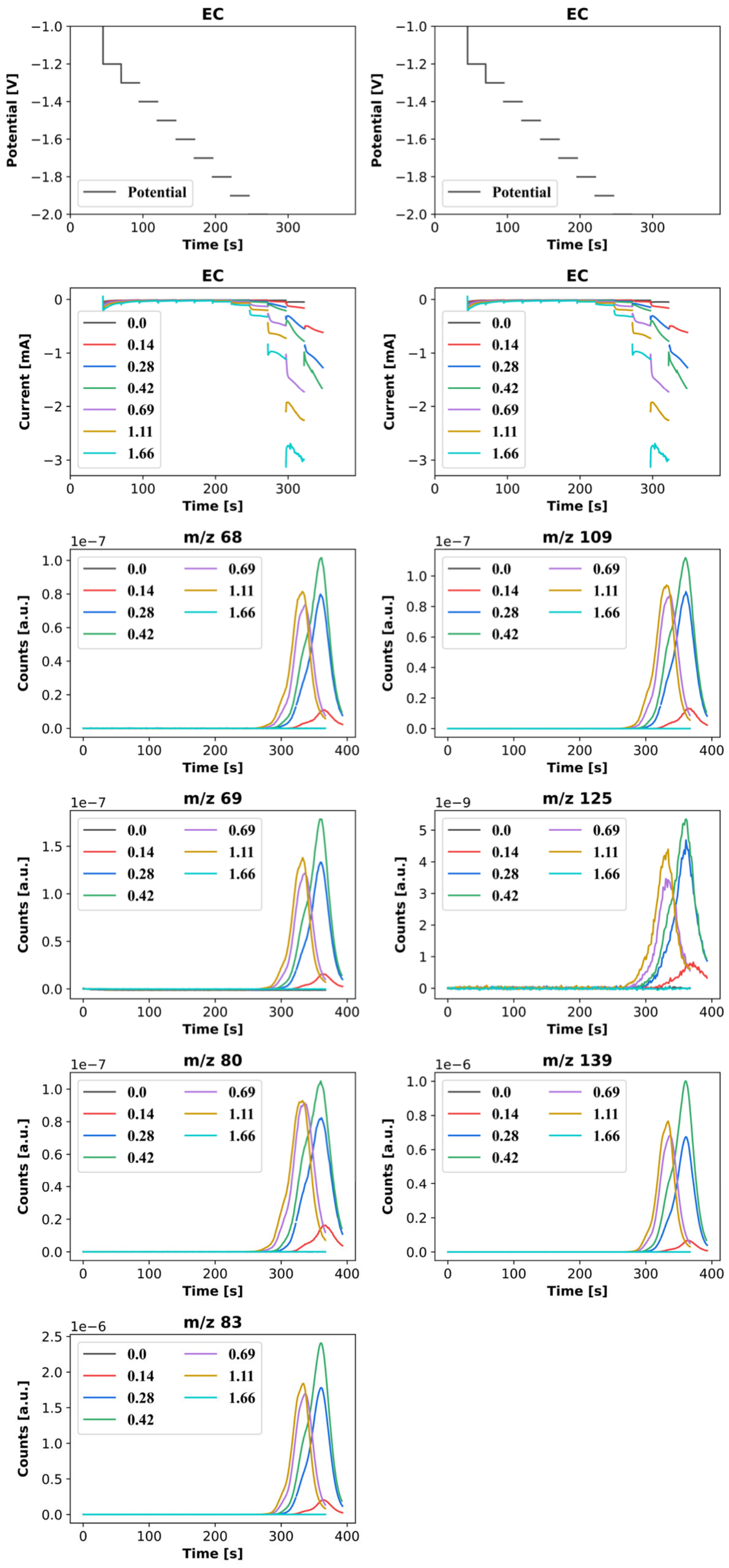

Figure S12d: *Observed mass charge ratios from 68 to 139 for the CA measurements on gold for the cathodic potentials. Background was subtracted.*

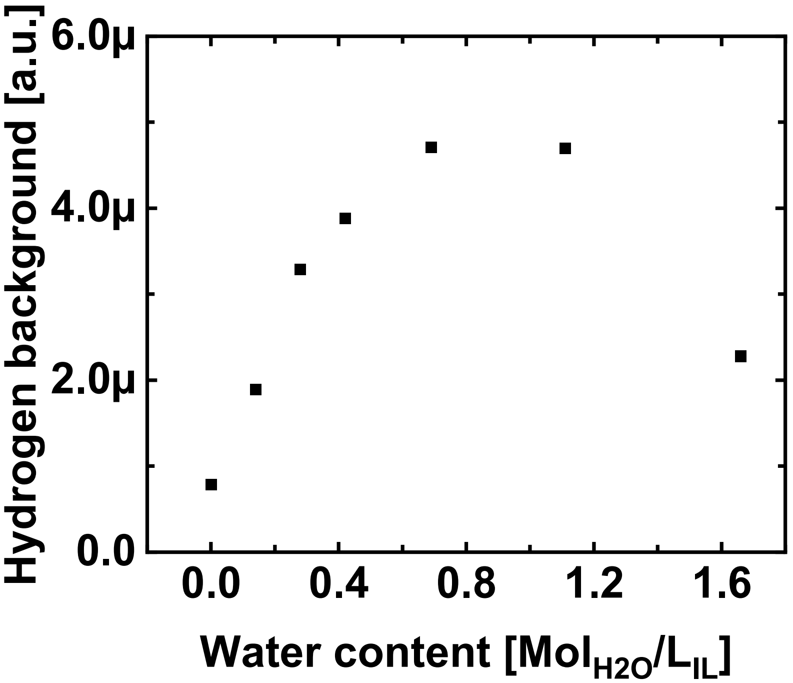

Figure S13: Hydrogen background extracted from the cathodic side of the platinum measurements in S7. The water content via the water signal in the DEMS could not be measured simultaneously due to the high value in comparison to the other signals. This would not have allowed for the sensitive measurements using a channel plate detector. However,, we believe, that the hydrogen background signal may be an indicator for the water content. The increase can be explained by the partial dissociation of the water during ionisation in the mass spectrometer. Figure S11 showed an increase of hydrogen background signal up until 0.8 M_{H2O}/L_{IL}. At higher water contents the signal dropped again. We believe, that at this point, the water vapor in the DEMS was so high, that the mean free path between the ionization and detection was enough influenced to change the signal strength. This needs to be considered for all signals. We therefore advise to analyze changes in the signals starting from 0.8 M_{H2O}/L_{IL} only qualitatively and not quantitatively. We also observed, that the start of flow influenced the total background pressure in the prechamber increasingly stronger with increasing water content supporting our hypothesis.

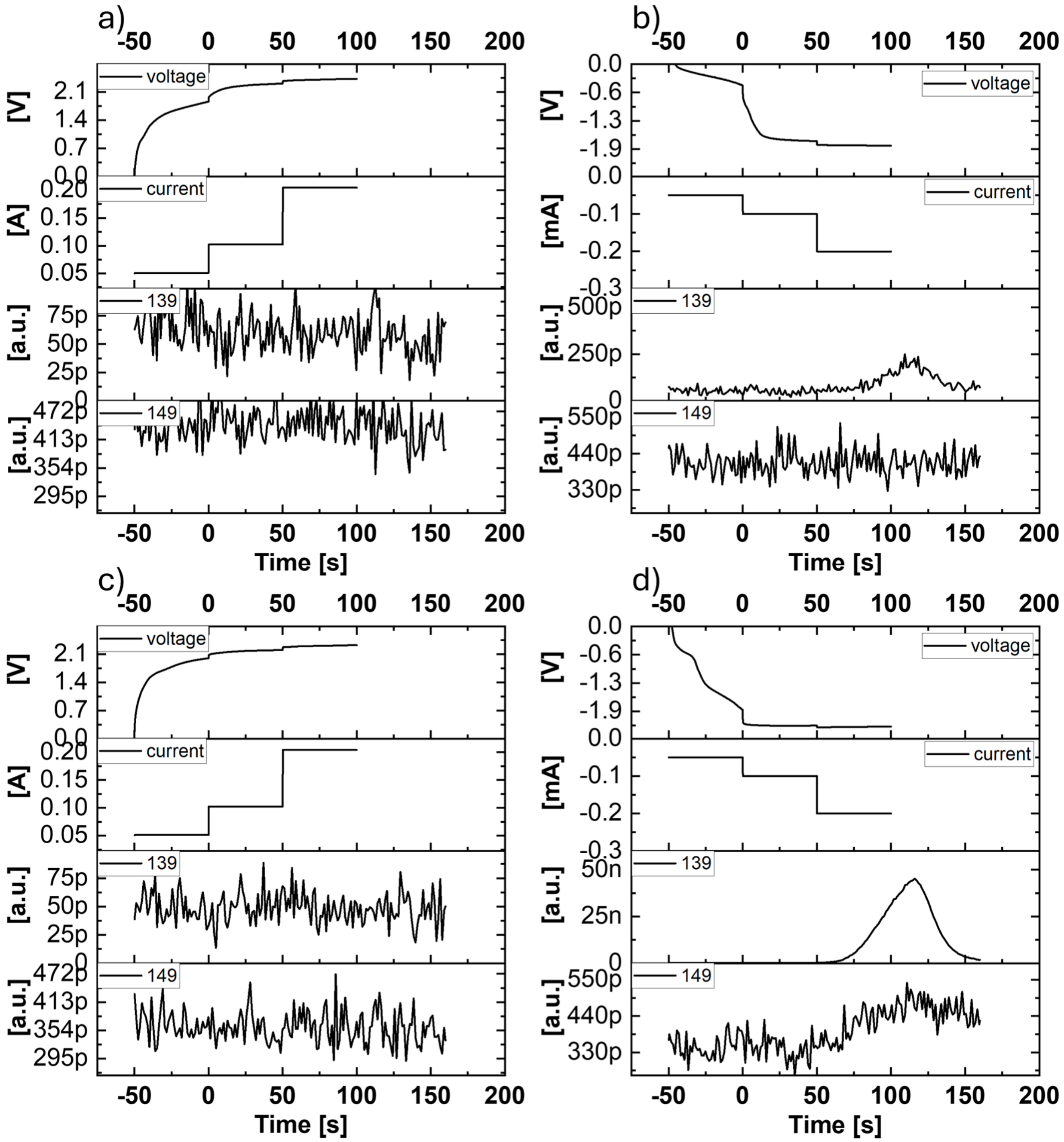

Figure S14: *CP measurements investigating the BMIM (139) and OTf (149) molecules for the (a,c) anodic and (b,d) cathodic ECW side on (a,b) platinum and (c,d) gold.*

Supporting Tables

Table S1: *Steps of added amounts of water to the 3 ml Ionic liquid in the glass cell.*

3 ml IL	BMIM Triflate		
Added Water [ml]	Volume % [v/v%]	Mol_{H2O}/L_{IL} [Mol/L]	H_2O/IL [Mol%]
0.000	0.000	0.000	0.000
0.020	0.662	0.369	0.082
0.040	1.316	0.738	0.164
0.060	1.961	1.107	0.245
0.080	2.597	1.476	0.327
0.100	3.226	1.845	0.409
0.150	4.762	2.767	0.614
0.200	6.250	3.690	0.818
0.300	9.091	5.534	1.227
0.500	14.286	9.224	2.045
0.700	18.919	12.913	2.864
1.000	25.000	18.448	4.091
1.500	33.333	27.671	6.136
2.000	40.000	36.895	8.182
2.500	45.455	46.119	10.227
3.000	50.000	55.343	12.273
5.000	62.500	92.238	20.455

Table S2. *Steps of added amounts of water to the 3 ml Ionic liquid in the DEMS cell.*

20 ml IL	BMIM Triflate		
Added Water [ml]	Volumen % [v/v%]	Mol_{H2O}/L_{IL} [Mol/L]	H_2O/IL [Mol%]
0.00	0.000	0.000	0.000
0.05	0.249	0.138	0.031
0.10	0.498	0.277	0.061
0.15	0.744	0.415	0.092
0.25	1.235	0.692	0.153
0.40	1.961	1.107	0.246
0.60	2.913	1.660	0.368

Table S3. *Detected mass-charge-ratios and possible fragments.*

m/z	possible compound	origin	Gold	Platinum
31	CF/CNH5	Anion or cation	Anodic and Cathodic	Anodic and Cathodic (very low signal)
39	C3H3	Cation (butyl group)	---	Anodic and Cathodic (very low signal)
41	C3H5	Cation (butyl group)	---	Anodic
42	C3H6	Cation (butyl group)	Anodic and Cathodic	---
43	C3H7	Cation (butyl group)	Anodic and Cathodic	---
44	CO2/C2H6N	Cation or carbon compound on surface reacting with O_2 from water or anion	Anodic and Cathodic	Anodic
48	SO	Anion	Anodic and Cathodic	Anodic
50	CF2	Anion	Anodic and Cathodic	Anodic
64	SO2	Anion	---	Anodic
68	C3N2H4/C4H6N	Cation	Cathodic	---
69	CF3/ C3H5N2	Anion or Cation	Anodic and Cathodic	Anodic
72	C4H10N/C3H8N2	Cation (Ring fragmentation)	---	Anodic and Cathodic(weak signal)
80	SO3	Anion	Cathodic	Not detected
83	C3N2CH7	Cation (Ring fragmentation)	Cathodic	---
99	SO3F	Anion	---	Anodic
109	C6H9N2	Cation	Cathodic	---
125	C6H13N2/CF3SO2	Cation or Anion	Cathodic	Cathodic (very low signal)
139	BMIM	Cation after loss of Anion	Cathodic	---
